# Health system impacts of SARS-CoV − 2 variants of concern: a rapid review

**DOI:** 10.1186/s12913-022-07847-0

**Published:** 2022-04-23

**Authors:** Justine Dol, Leah Boulos, Mari Somerville, Lynora Saxinger, Alexander Doroshenko, Stephanie Hastings, Bearach Reynolds, Allyson Gallant, Hwayeon Danielle Shin, Helen Wong, Daniel Crowther, Marilyn Macdonald, Ruth Martin-Misener, Holly McCulloch, Andrea C. Tricco, Janet A. Curran

**Affiliations:** 1 Faculty of Health, Dalhousie University, Halifax, NS Canada; 2 Maritime SPOR SUPPORT Unit, Halifax, NS Canada; 3 School of Nursing, Dalhousie University, Halifax, NS Canada; 4 Division of Infectious Diseases, Departments of Medicine and Medical Microbiology and Immunology, University of Alberta, Edmonton, AB Canada; 5 Division of Preventive Medicine, Faculty of Medicine and Dentistry, University of Alberta, Calgary, AB Canada; 6 Alberta Health Services, Calgary, AB Canada; 7 Evidence Synthesis, Galway, Ireland; 8 IWK Health, Halifax, NS Canada; 9 Knowledge Translation Program, Li Ka Shing Knowledge Institute, St. Michael’s Hospital, Unity Health, Toronto, ON Canada; 10 Epidemiology Division and Institute for Health Policy, Management, and Evaluation, Dalla Lana School of Public Health, University of Toronto, Toronto, ON Canada; 11 Queen’s Collaboration for Health Care Quality Joanna Briggs Institute Centre of Excellence, School of Nursing, Queen’s University, Toronto, ON Canada

**Keywords:** SARS-CoV − 2, Variants of concern, Health system impact, Rapid review

## Abstract

**Background:**

As of November 25th 2021, four SARS-CoV − 2 variants of concern (VOC: Alpha (B.1.1.7), Beta (B.1.351), Gamma (P.1), and Delta (B.1.617.2)) have been detected. Variable degrees of increased transmissibility of the VOC have been documented, with potential implications for hospital and health system capacity and control measures. This rapid review aimed to provide a synthesis of evidence related to health system responses to the emergence of VOC worldwide.

**Methods:**

Seven databases were searched up to September 27, 2021, for terms related to VOC. Titles, abstracts, and full-text documents were screened independently by two reviewers. Data were extracted independently by two reviewers using a standardized form. Studies were included if they reported on at least one of the VOC and health system outcomes.

**Results:**

Of the 4877 articles retrieved, 59 studies were included, which used a wide range of designs and methods. Most of the studies reported on Alpha, and all except two reported on impacts for capacity planning related to hospitalization, intensive care admissions, and mortality. Most studies (73.4%) observed an increase in hospitalization, but findings on increased admission to intensive care units were mixed (50%). Most studies (63.4%) that reported mortality data found an increased risk of death due to VOC, although health system capacity may influence this. No studies reported on screening staff and visitors or cohorting patients based on VOC.

**Conclusion:**

While the findings should be interpreted with caution as most of the sources identified were preprints, evidence is trending towards an increased risk of hospitalization and, potentially, mortality due to VOC compared to wild-type SARS-CoV − 2. There is little evidence on the need for, and the effect of, changes to health system arrangements in response to VOC transmission.

**Supplementary Information:**

The online version contains supplementary material available at 10.1186/s12913-022-07847-0.

## Background

The World Health Organization (WHO) declared a global pandemic from the SARS-CoV − 2 virus, responsible for COVID-19, in March 2020 [1]. Over 246 million cases of COVID-19 had been reported worldwide along with 5 million deaths [2]. The continued rise in COVID-19 cases is causing grave concerns on the threatened capacity of health systems to manage current and new admissions for COVID-19 while still providing sufficient care on all other health conditions. This situation has been made more acute by the emergence of variants of concern (VOC).

As of mid-November 2021, four variants of the original SARS-CoV − 2 lineage (i.e., wild-type) have been declared as a VOC by the WHO, with other variants of interest being continuously monitored [3]. According to the WHO, VOC are defined by their increased potential for transmission or changes in COVID-19 epidemiology, presence of genomic mutations, and rapid spread across countries or regions, possibly leading to the decreased effectiveness of public health measures or of diagnostic tests, vaccines, and therapeutics [4, 5]. Variants of concern may have a transmission advantage which, if present, over time will lead to replacement of circulating strains with new VOC [6]. Public health and hospital-based interventions and control measures in these circumstances may need to focus on the growth of more transmissible variants, rather than total numbers of cases.

In December 2020, the variants Alpha (B.1.1.7, identified in the United Kingdom [UK]) and Beta (B.1.351, identified in South Africa) were named the first VOC by the WHO, followed by Gamma (P.1, identified in Brazil) in January 2021, and Delta (B.1.617.2, identified in India) in May 2021 [5]. Data indicates that Alpha is associated with a 43-90% increased risk of transmission compared to wild type, [7–9], and Beta is between 1.5 [10, 11] and 2.5 [8] times more transmissible. Delta is estimated at 60% more transmissible than Alpha [12]. Trends suggest that all VOC to date have a transmission advantage over wild-type [6–8].

The increased transmissibility of VOC has led to surges in COVID-19 incidence and, consequently, more hospitalizations and higher mortalities in some areas [9]. The first wave of the pandemic demonstrated the potential for even well-equipped health systems to experience overwhelmed intensive care units (ICUs) and system disruption, with wide ranging health consequences [13]. Furthermore, due to the rapid and emergent nature of SARS-CoV − 2 and VOC, health systems and public health administrators have been challenged to make pragmatic decisions in the absence of evidence. With health systems continuously under stress as a result of changes to public health restrictions [14], having to address increased waitlists from restricted access to care, and the introduction of new VOC, there is an ever growing need to optimize management of VOC patients to reduce risk and maintain capacity.

Therefore, this rapid review aimed to provide a synthesis of current evidence related to health system impacts in the context of VOC. This review is part of a larger review on transmission [6] and public health impacts [15]. The objective of this rapid review was to identify, appraise, and summarize evidence about health system impacts of the four major WHO-defined SARS-CoV − 2 VOC known as of May 2021 (Alpha, Beta, Gamma, and Delta). Based on iterative knowledge user and shareholder meetings, the following questions were derived:

What is known about the implications of the WHO-defined VOC for health system arrangement (particularly for hospitals) on:

- Adjusting capacity planning to accommodate changes in the risk of re-infection and the risk of severe disease (e.g., hospitalization, admission to ICU, and death)
- Adjusting personal protective equipment (PPE) procedures for health workers
- Adjusting restrictions and screening of staff and visitors (e.g., visitor policy changes, approach to and frequency of screening)
- Adjusting service provision (e.g., cohorting patients in hospitals based on the VOC they have acquired)
- Adjusting patient accommodations, shared spaces, and common spaces (e.g., improvement to HVAC [heating, ventilation, and air conditioning systems])

## Methods

### Design

We conducted a rapid review following standardized rapid methodological guidelines [16, 17]. We used an integrated knowledge translation approach, as the question was initially designed by knowledge users and refined with the synthesis team with continuous exchange during the process through regular meetings. The knowledge user partners, who are health system and infectious disease experts, reviewed the results. Patient partners were engaged in the knowledge dissemination phase to provide feedback on the final report and provide recommendations from the patient perspective.

#### Protocol

A protocol was developed using Joanna Briggs Institute (JBI) guidance [18] and reported according to the Preferred Reporting Items for Systematic Reviews (PRISMA) for Protocols [19]. The protocol is available on Open Science Framework [20]. The results are reported using the PRISMA 2020 guidelines [21].

#### Literature search

A broad, comprehensive literature search was designed by an information specialist to retrieve all literature related to VOC. The electronic database search was executed on May 11, 2021 and updated on September 27, 2021 in MEDLINE (Ovid MEDLINE All), Embase (Elsevier Embase.com), the Cochrane Database of Systematic Reviews (CDSR) and Central Register of Controlled Trials (CENTRAL) (Cochrane Library, Wiley), Epistemonikos’ Living Overview of Evidence (L·OVE) on COVID-19, and medRxiv and bioRxiv concurrently. The MEDLINE, Embase, and Cochrane Library searches used modified versions of COVID-19 filters developed by the Canadian Agency for Drugs and Technology in Health (as they appeared at the time of search development in February 2021) [22]. The search was peer reviewed by a second information specialist using the Peer Review of Electronic Search Strategies (PRESS) guideline [23]. Full search details are available as Supplementary Material for all databases.

#### Eligibility criteria

All studies that reported on health system impacts due to VOC were included. Studies that reported on immune escape (vaccine/prior infection protection), non-VOC related impacts, testing approaches, transmission or public health impacts, case studies without health system impacts, or animal studies were excluded. Reviews, overviews, and news articles that presented no original data were excluded, but references were scanned to identify additional relevant studies. Only English-language searches were conducted, but non-English results were considered for inclusion.

#### Screening and data extraction process

After a pilot-test exercise amongst the team, titles/abstracts and full-text screening was completed by two reviewers in Covidence. The data extraction form was designed in consultation with knowledge user partners and pilot-tested amongst the team. Data extraction was completed by two reviewers and verified by a third.

#### Critical appraisal

Critical appraisal for observational studies was conducted using the Joanna Briggs Institute (JBI) appraisal tools [18]. Two team members independently conducted critical appraisals for all eligible studies. Reviewers met to discuss scores, and a third, independent team member was consulted to assist with resolving conflicts. Modeling studies and lab-based studies were not appraised due to the absence of a standardized appraisal tool for these study types. As the quality of preprints should be interpreted with caution, efforts were made to reflect this through the removal of two points from the overall score. Similarly, one point was removed from any published letters to the editor as they are not fully peer reviewed, yet they are published in a peer reviewed journal. Cohort studies were awarded a maximum of 11 points, case control studies awarded a maximum of 10 points, and cross-sectional studies were awarded a maximum of eight points. Final scores for observational studies were presented as a percentage, based on an average between the two appraiser scores. An overall quality rating of low, medium, or high was reported for each observational study, which correlated with a score of < 50%, 50-80% or > 80% respectively.

#### Synthesis

The results were presented descriptively in text, tables, and diagrams. A meta-analysis was not possible due to heterogeneity across the included studies regarding their study designs, participants included, and VOC.

## Results

**Fig. 1:**
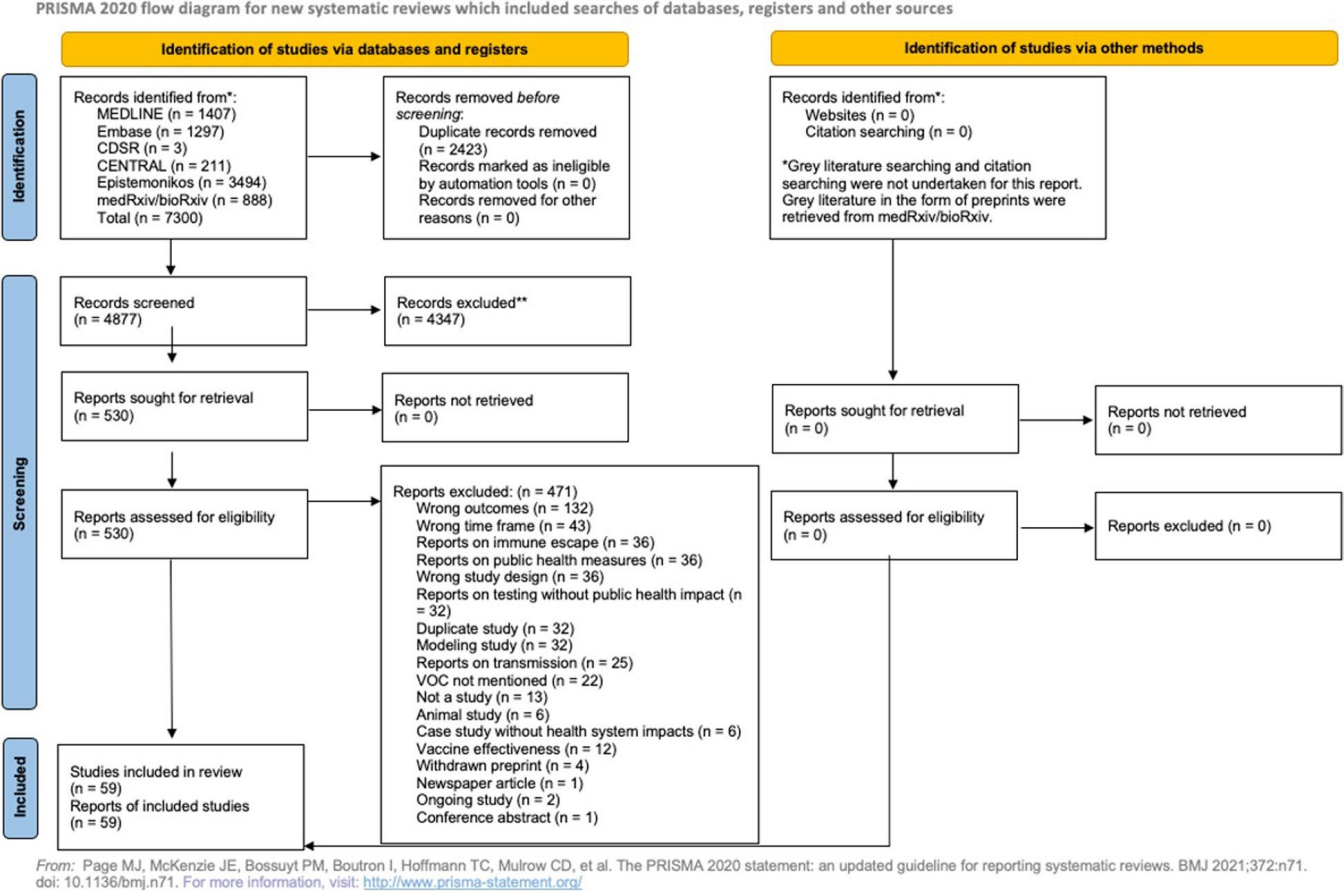
PRISMA flow diagram

**Fig. 2:**
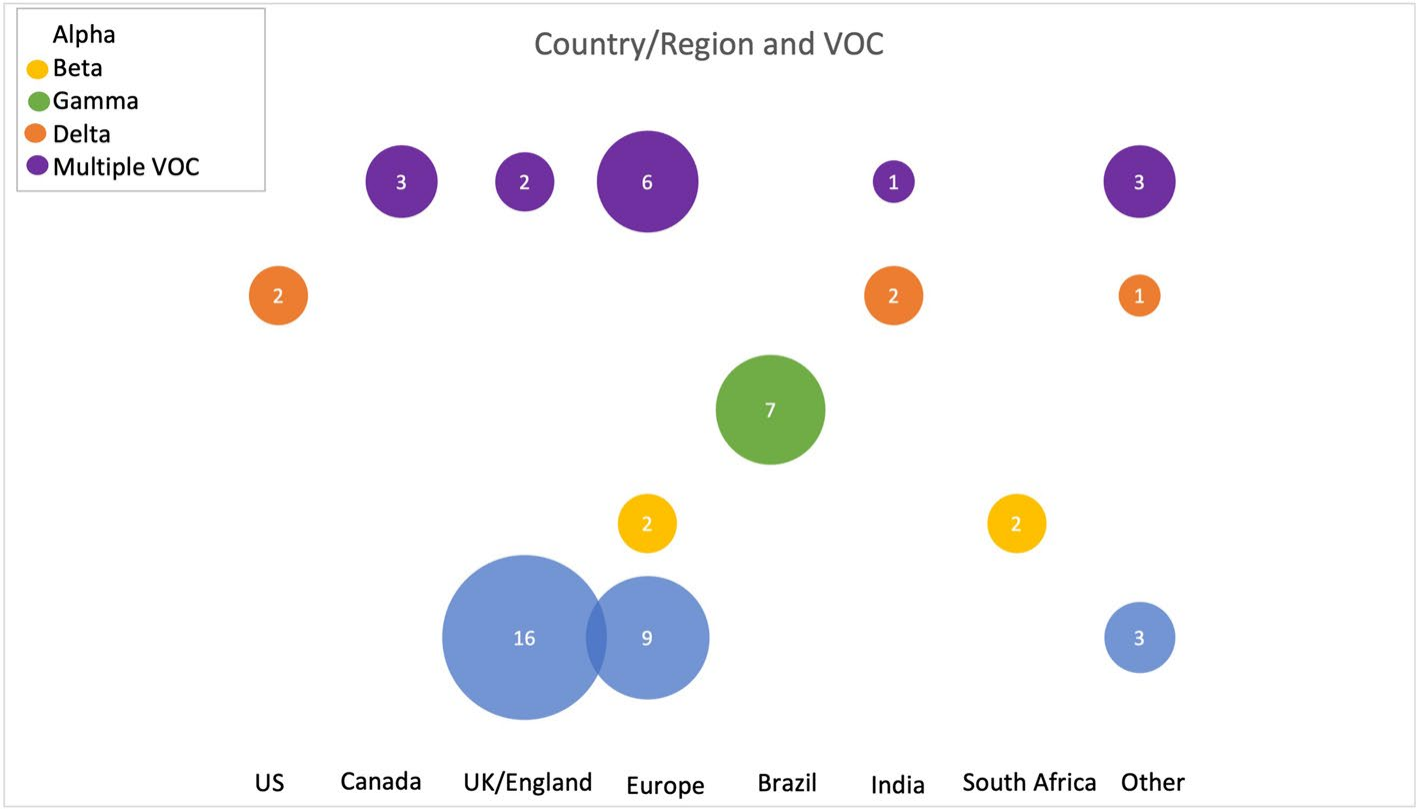
Overview of country or region of data collection and VOC up to September 27, 2021

**Fig. 3:**
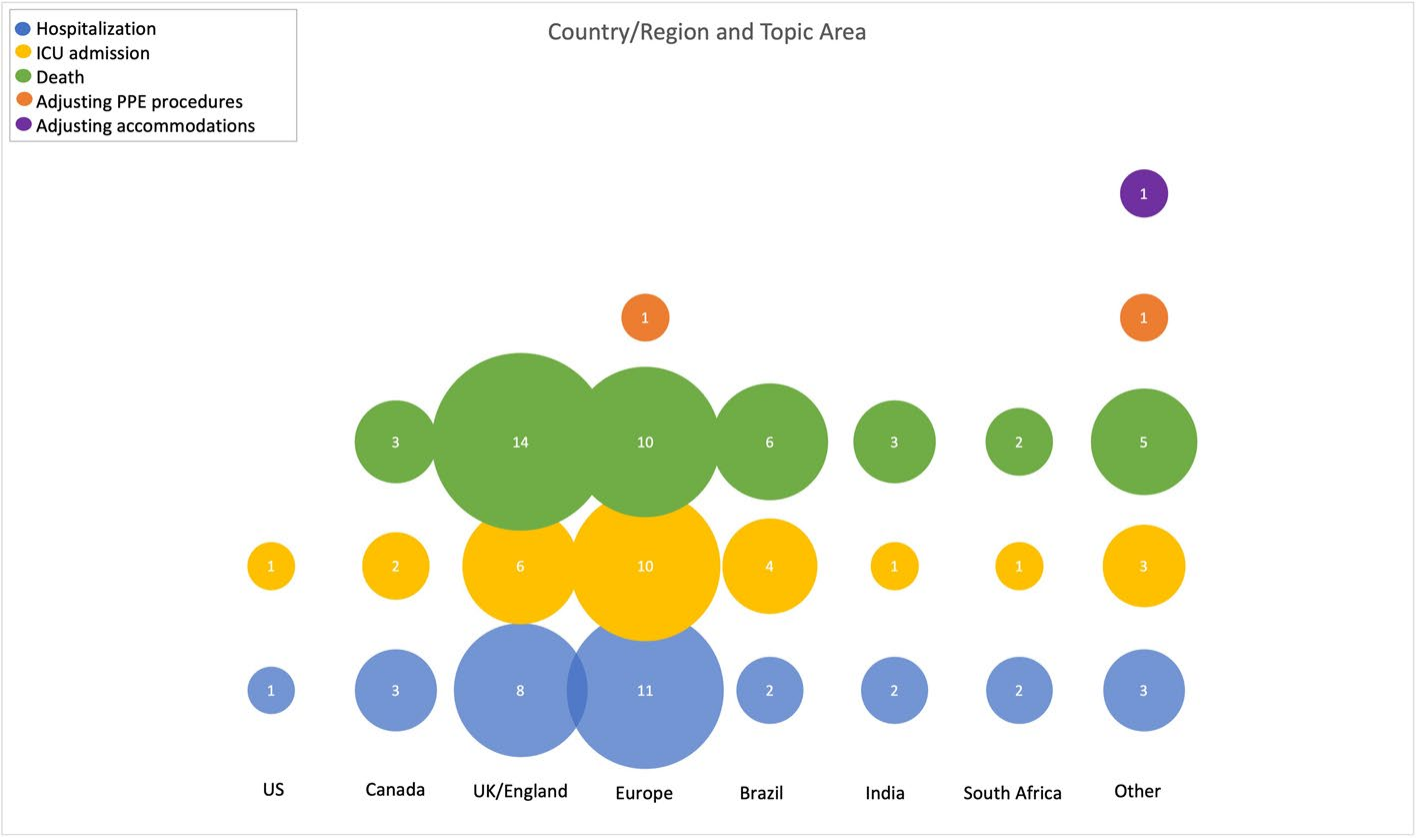
Overview of country or region of data collection and outcome up to September 27, 2021

The search identified 7300 records; 4877 records were screened after duplicate removal using Covidence, and 59 studies that reported on health system impacts were included (25 identified in the search on May 11, 2021, and 34 identified on September 27, 2021) (see Fig. 1 for PRISMA Flow Diagram). Of note, the search was intended to be very broad due to the significant variation in reporting and terminology in early VOC literature. In total, 25 preprints and 34 peer-reviewed journal articles were identified (see Supplementary Material 2 for a summary table of included studies). In the updated search, six studies that were originally included as preprints had subsequently been published in a peer reviewed journal. Alpha was the most reported-on VOC (*n* = 28). Seven studies reported on Gamma, four studies reported on Beta, five studies reported on Delta, and fifteen studies reported on multiple VOC. Most of the studies were from the UK or England (*n* = 18), followed by Brazil (*n* = 7) and France (*n* = 6). Three studies reported on multiple European countries. Figure 2 provides an overview of country or region of data collection and VOC up to September 27, 2021, while Fig. 3 provides an illustration of the number of studies on each of the outcomes from all countries.

### Critical appraisal

Of the 59 studies, 31 were cohort studies, 20 used a cross-sectional design, and one was a case control study; thus, they were subject to appraisal using the relevant JBI checklists. Among the 51 cohort/cross-sectional studies, five were appraised as low quality [24–28], 24 as medium quality [29–52], and 22 as high quality [53–74]. The case control study was of medium quality [75]. A complete overview of JBI scores by study can be found in Table 1. Of note, six were modeling studies and one was a lab-based study; these were therefore not included in the quality assessment.

**Table 1:** Critical Appraisal research articles using the Joanna Briggs Institute (JBI) checklists (High quality: 80-100%; Medium quality: 50-80%; Low quality: < 50%)

Author, year	Pre-print (PP)/ Peer Review (PR)	Source	Average score	Adjust for PP or LE	Total Score (%)	Overall Quality
**Cohort Study Design**^**a**^						
Bager, 2021	PR	Lancet	9.5	N/A	9.5 (86)	High
Budhiraja, 2021	PP	MedRxiv	7.5	−2	5.5 (50)	Medium
Challen, 2021	PR	BMJ	10.5	N/A	10.5 (95)	High
Cusinato, 2021	PR	Infection	10.5	N/A	10.5 (95)	High
Dabrera, 2021	PP	SSRN	9.5	−2	7.5 (68)	Medium
Dennis, 2021	PR	Critical Care Medicine	9.5	N/A	9.5 (86)	High
Erman, 2021	PP	MedRxiv	9.5	−2	7.5 (68)	Medium
Fisman, 2021	PP	MedRxiv	9	−2	7 (64)	Medium
Frampton, 2021	PR	Lancet	10.5	N/A	10.5 (95)	High
Freitas B, 2021	PP	MedRxiv	8	−2	6 (55)	Medium
Grint, 2021	PR	Clinical Infectious Diseases	10.5	N/A	10.5 (95)	High
Haas, 2021	PR	Lancet	10	N/A	10 (91)	High
Havers, 2021	PP	MedRxiv	10.5	−2	8.5 (77)	Medium
Jassat, 2021	PR	Lancet Global Health	10.5	N/A	10.5 (95)	High
Khedar, 2021	PP	MedRxiv	9	−2	7 (64)	Medium
Martin-Blondel, 2021	PP	SSRN	8.5	−2	6.5 (59)	Medium
Maslo, 2021	PP	MedRxiv	9.5	−2	7.5 (68)	Medium
McAllister, 2021	PP	MedRxiv	9	-2	7 (64)	Medium
Nyberg, 2021	PR	BMJ Open	11	N/A	11 (100)	High
Ong, 2021	PR	Clinical Infectious Diseases	10	N/A	10 (91)	High
Pascall, 2021	PP	MedRxiv	10	-2	8 (73)	High
Patone, 2021	PR	Lancet	9.5	N/A	9.5 (86)	High
Peuch, 2021	PP	Research Square	10.5	-2	8.5 (77)	Medium
Stirrup, 2021	PR	BMJ Open Respiratory Research	11	N/A	11 (100)	High
Swann, 2021	PP	MedRxiv	10	-2	8 (73)	Medium
Twohig, 2021	PR	Lancet Infectious Diseases	9.5	N/A	9.5 (86)	High
Vassallo, 2021	PR	Journal of Clinical Medicine	8.5	N/A	8.5 (77)	Medium
Veneti A, 2021	PR	PLOS One	9.5	N/A	9.5 (86)	High
Veneti B, 2021	PP	MedRxiv	9	-2	7 (88)	High
Whittaker, 2021	PR	Journal of Infection	9	N/A	9 (82)	High
Zavaski, 2021	PP	Research Square	9.5	-2	7.5 (68)	Medium
**Cross-sectional Study Design**^**b**^						
Adhikari, 2021	PR	American Journal of Obstetrics & Gynecology	4	N/A	4 (50)	Medium
Agrawal, 2021	PR	European Journal of Molecular & Clinical Medicine	5	N/A	5 (63)	Medium
AlQahtani, 2021	PP	Research Square	7	-2	5 (63)	Medium
Area, 2021	PR	International Journal of Environmental Research and Public Health	7	N/A	7 (88)	High
Cetin, 2021	PR	Acta Microbiologica et Immunologica Hungarica	5.5	N/A	5.5 (69)	Medium
Courjon, 2021	PR	Nature	6	N/A	6 (75)	Medium
de Andrade, 2021	PP	MedRxiv	3.5	-2	1.5 (19)	Low
de Oliveira, 2021	PP	MedRxiv	3	-2	1 (13)	Low
Freitas A, 2021	PP	SciELO pre-prints	5.5	-2	3.5 (44)	Low
Funk, 2021	PR	Eurosurveillance	7	N/A	7 (88)	High
Garvey, 2021	LE	Journal of Infection	7.5	−1	6.5 (81)	High
Graham, 2021	PR	Lancet	7	N/A	7 (88)	High
Jablonska, 2021	PP	MedRxiv	5.5	−2	3.5 (44)	Low
Loconsole, 2021	PR	Environmental Research and Public Health	5	N/A	5 (63)	Medium
Louis, 2021	LE	Anaesthesia Critical Care & Pain Medicine	5.5	−1	4.5 (56)	Medium
Martinez-Garcia, 2021	PR	Microorganisms	6	N/A	6 (75)	Medium
Moore, 2021	PP	MedRxiv	3	−2	1 (13)	Low
Nonaka, 2021	PR	International Journal of Infectious Diseases	6.5	N/A	6.5 (81)	High
Snell, 2021	PP	MedRxiv	6	−2	4 (50)	Medium
Takemoto, 2021	PP	MedRxiv	6	−2	4 (50)	Medium
**Case Control Study Design**^**c**^						
Abu-Raddad, 2021	PR	Clinical Infectious Diseases	7.5	N/A	7.5 (75)	Medium

LE: letter to editor; Scored out of 11^a^; Scored out of 8^b^; Scored out of 10^c^

### Question A: Adjusting capacity planning to accommodate changes in the risk of re-infection and the risk of severe disease (e.g., hospitalization, admission to ICU, and death)

Figure 3 provides an overview of studies that explored various aspects of capacity planning in relation to country or region. While most studies related to this sub-question reported on the impact of VOC on hospitalization, admission to ICU, and mortality, six studies reported on outcomes in relation to vaccines. Haas et al. [59] conducted the first nationwide estimates on the vaccine effectiveness (VE) of two doses of the Pfizer vaccine on hospitalization and deaths in Israel during a period of high Alpha prevalence. They found that adjusted VE against COVID-19 hospitalization was 97.2% (95% CI 96.8-97.5), against severe and critical hospitalization was 97.5% (95%CI: 97.1-97.8), and against death was 96.7% (95%CI: 96.0-97.3). AlQahtani et al. [51] compared four vaccines (Astra-Zeneca, Pfizer/BioNtech, Sinopharm, and Sputnik V) and found that all were effective in decreasing risk of hospitalization, ICU admission, and mortality prior to and during the period when Delta was dominant, although the Sinopharm vaccine is less effective than the Pfizer/BioNtech on all outcomes. Agrawal et al. [40] found that in patients with pre-existing medical conditions who were infected with Delta but vaccinated were less likely to die than patients who were unvaccinated (*p* = 0.002); no difference was found in patients without pre-existing conditions who were infected with either Alpha or Delta. Havers et al. [42] found that hospitalization rates were > 10 times higher in unvaccinated individuals compared to vaccinated individuals during a period of high Delta prevalence. Twohig et al. [70] found that patients with Delta who were unvaccinated or < 21 days since first dose had a higher estimated risk of hospital admission and a higher risk of either hospital admission or emergency care than patients with Alpha; however, there was no significant interaction when comparing between vaccinated and unvaccinated individuals infected with either Delta or Alpha. Veneti et al. [72] found that after adjusting for sex, age group, country of birth, variant and underlying comorbidities, partially vaccinated individuals had a 72% reduced risk of hospitalization (95%CI 59–82%) and fully vaccinated had a 76% reduced risk of hospitalization (95%CI 61–85%) compared to unvaccinated individuals with Delta or Alpha.

Di Domenico et al. also reported on outcomes differently, in that they provided an age-stratified transmission model to estimate the role that curfew measures could have on hospitalization in France [76]. They found that if the epidemic progressed under curfew conditions (6:00 pm nightly, implemented nationwide January 16, 2021) before school holidays and vaccination was accelerated, hospital capacity would be reached around week 13 in France (which had 2.2% Alpha penetration), week 12 in Île-de-France (which had the highest Alpha penetration, 6.9%), and week 14 in Nouvelle Aquitaine (which had the lowest Alpha penetration, 1.7%). This was supported by data. The partial relaxation of social distancing (estimated at a 15% increase in effective reproduction number) would shorten these estimates by at least one week. Stronger social distancing, equivalent to the efficacy measured during the second lockdown (estimated at a 15% reduction in effective reproduction number), would maintain hospitalizations below the peak of the second wave in Île-de-France and Nouvelle Aquitaine but would not be enough to avoid a third wave in France, even under accelerated vaccination (100,000 − 200,000 doses/day). Accelerated (200,000 first doses/day) and optimistic vaccination rollouts (300,000 first doses/day) would reduce weekly hospitalizations by about 20 and 35% in week 16 (i.e., April 19 − 25, 2021) compared to a stable vaccination campaign without acceleration (100,000 first doses/day).

Finally, Ong et al. [66] reported on a composite measure of disease severity, defined by a composite outcome of oxygen requirement, ICU admission, and death, and found that Delta was associated with increased disease severity compared to non-VOC (unadjusted OR 5.55 (95% 1.66 – 34.44); adjusted OR 4.90 (95%CI 1.43 – 30.78)). No difference was found for Alpha or Gamma.

#### Impact of VOC on hospitalization/severity

Thirty-one studies reported on health system impacts related to hospitalization and/or severity of disease (see Table 2). Of the four VOC, Alpha was the most predominantly reported on, with fourteen studies finding an increase in hospitalization due to Alpha, while five studies reported no change. Fewer studies reported on the other VOC: five studies on Beta (three with increases in hospitalization and two with no change), two studies on Gamma (two reported increases in hospitalization) and four studies on Delta (two reported increases and two no change). Four studies reported on combined VOC, with all studies finding an increase in hospitalization compared to non-VOC. Overall, 73.5% of studies reported increases in hospitalization and/or severity due to any VOC compared to non-VOC.

**Table 2:** Summary of findings related to hospitalization and severity of illness

VOC	Increased hospitalization/severity due to VOC	No change in hospitalization/severity due to VOC
**Alpha**	• No significant difference was found in hospitalization between Alpha vs. non-Alpha in the crude analysis (RR 0.79, 95% CI 0.72–0.87), but after adjusting for sex, age, region, and comorbidities, Alpha was 1.4 times more likely to be associated with hospitalization than wild type (adjusted RR 1.42; 95% CI 1.25–1.60) (Bager et al., Denmark, Jan-Feb 2021, medium quality )[53] • In wave two (high Alpha prevalence), the number of admissions increased (35.1% vs. 54.8%) from wave one (non-Alpha). Patients with non-Alpha and Alpha were not significantly different in terms of age or ethnicity. Alpha patients were more likely to be female (48.0% vs 41.8%, *p* = 0.01), less likely to be frail (14.5% vs 22.4%, *p* = 0.001), and more likely to be obese (30.2% vs 24.8%, *p* = 0.048) than non-Alpha patients. On admission, patients with Alpha were more likely to be hypoxic, which was the main indicator of severe disease (Snell et al., UK, Mar 2020-Feb 2021, medium quality )[41] • There was a non-significant association between infection with Alpha and hospitalization within 14 days of a positive test (OR 1.39, 95% CI: 0.98-1.98, *p* = 0.07). In a univariable analysis, Alpha infection and risk of hospitalization within 14 days were not associated (Hazard Ratio (HR) 1.07, 95% CI 0.89-1.29, *p* = 0.48); however, adjusting for potential confounders (sex, age, ethnicity, residential property classification, and week of specimen date) suggested a higher risk of hospitalization from Alpha (HR: 1.34, 95% CI 1.07-1.66, *p* = 0.01). (Dabrera et al., UK, Oct-Dec 2020, medium quality)[31] • Individuals with Alpha (SGFT-positive) were more likely to be hospitalized (OR 3.44, 95% CI 1.76-6.75) than non-Alpha (SGFT-negative) cases (Loconsole et al., Italy, Dec 2020-Mar 2021, medium quality )[48] • Alpha was associated with 62% increased risk of hospital admission (aHR: 1.62; 95% CI: 1.48–1.78; *P* < .0001) compared with wild-type. Among people admitted to a hospital, those with Alpha were younger (median [IQR] age: 57.0 [47.0–68.0] vs 59.0 [48.0–72.0] years) and had fewer comorbidities (≥2 comorbidities: 19.3% vs 25.2%) compared with those with wild-type (Grint et al., England, Nov 2020-Jan 2021, high quality)[58] • Compared to wild-type, the odds of progressing to severe disease were 1.48-fold (95% CI 1.18-1.84) higher for Alpha (Abu-Raddad, Qatar, Jan-May 2021, medium quality)[75] • In the Alpha wave, most hospitalizations were in people over 80, but there was also an increase in hospitalizations in children aged 10 and older (0.21% to 0.35%) compared to previous waves (Area et al., Spain, March-April 2021, high quality)[62] • Alpha increased the chance of hospitalization: 13% of the outpatients had Alpha vs. 33% of inpatients (Cetin et al., Turkey, April 2020-March 2021, medium quality)[46] • The number of COVID-19 admissions was 2.05 times higher in the Alpha wave compared to the pre-Alpha wave. COVID-19 patients admitted during the Alpha wave were more likely to be younger with intermediate levels of frailty (Cusinato et al., UK, January 2020-March 2021, high quality)[55] • In patients hospitalized with COVID-19, Alpha was associated with a 33% higher risk of severe COVID-19 than wild-type (aOR 1.33 95% CI 1.03-1.72) (Martin-Blondel, France, Jan-Feb 2021, medium quality)[34] • Individuals infected with VOC, primarily Alpha, were more likely to require hospitalization (aOR 1.57 [95% CI 1.47-1.69] in Ontario and aOR 1.88 [95% CI 1.74-2.02] in Alberta) than those without VOC (McAlister et al., Canada, March 2020-March 2021, medium quality)[35] • Risk of hospital admission within 14 days after a positive test was higher for patients with Alpha than wild-type (HR 1.52 (95% CI 1.47 to 1.57). The absolute risk of hospital admission after 14 days was 4.7% (95% CI 4.6 to 4.7%) for patients with Alpha and 3.5% (95% CI 3.4 to 3.5%) for those wild-type (Nyberg et al., England, Nov 2020-Jan 2021, high quality)[61] • Alpha was associated with more severe disease than those from other lineages (median cumulative odds ratio: 1.40, 95% CI 1.02-1.93) (Pascall et al., Scotland, Nov 2020-Jan 2021, high quality)[67] • Alpha was associated with a 1·9-fold increased risk of hospitalization compared to non-VOC (aRR 95%CI 1.6-2.3) (Veneti et al., Norway, Dec 2020-Jun 2021, high quality)[71]	• After correcting for mean age, sex, ambient temperature, and humidity, there was no association between Alpha and the number of symptoms reported over a 4-week period after a positive test or the number of hospitalizations (Graham et al., Scotland, Wales and England, Sep-Dec 2020, high quality )[65] • While risk of hospitalization within 14 days of a test and time to hospital admission from symptom onset were similar, Alpha patients were younger, had fewer comorbidities, and more likely to be from an ethnic minority compared to non-Alpha patients (Frampton et al., UK, Nov-Dec 2020, high quality)[57] • Pairing 29 Alpha cases to 58 controls (non-Alpha) on age and gender, there was no significant difference in time from first symptoms to emergency department admission or severity (Courjon et al., France, Dec 2020-Feb 2021, medium quality)[47] • Alpha did not lead to more severe disease in children and young people in the UK, with children admitted during the Alpha wave having lower Paediatric Early Warning Scores (PEWS) at presentation, lower antibiotic use, and less respiratory and cardiovascular support (Swann et al., UK, Jan 2020-Jan 2021, medium quality)[37] • There was no statistically significant difference between time from symptom onset to hospitalization or length of stay between Alpha patients and non-VOC patients (Whittaker et al., Norway, Dec 2020-Apr 2021, high quality)[73]
**Beta**	• Compared to Alpha, the odds of progressing to severe disease were 1.24-fold (95% CI 1.11-1.39) higher for Beta (Abu-Raddad, Qatar, Jan-May 2021, medium quality)[75] • Hospital admission rates were significantly higher in the second wave than the first (27.9 vs. 16.1 admissions per 100,000 people). The weekly average growth rate in hospital admissions was 20% in pre-Beta wave and 43% in Beta wave (ratio of growth rate was 1.19, 95% CI 1.18–1.20) (Jassat et al., South Africa, March 2020-March 2021, high quality)[60] • Beta was associated with a 2.4-fold increased risk of hospitalization compared to non-VOC (aRR 95%CI 1.7–3.3) (Veneti et al., Norway, Dec 2020-Jun 2021, high quality)[71]	• Similar amounts of patients were admitted to the hospital in the Beta wave compared to the pre-Beta wave (685 vs. 550), although patients admitted in the Beta wave were older and more likely to have no comorbidities (Maslo et al., South Africa, June-Dec 2020, medium quality)[44] • No differences for patients admitted to hospital with Beta or wild-type in terms of days between onset of symptoms (5 days) (Pascall et al., Scotland, Nov 2020-Jan 2021, high quality)[67]
**Gamma**	• There was an increase in proportion of patients with severe COVID-19, from 5% in the first wave to 10% in the second wave (associated with Gamma). There was no difference between sexes, but the proportion of patients with pre-existing conditions among severe cases was higher in the second wave (33%) compared to the first (25%), as well as higher proportion under age 60 (47% vs 39%) (Freitas et al., Brazil, Nov 2020-Feb 2021, medium quality)[28] • The incidence rate of advanced respiratory support (HR 1.78, 95% CI 1.05-3.03, *p* = 0.03) and invasive respiratory support (HR 2.64, 95% CI 1.34-5.19, *p* = 0.005) was higher in Gamma patients than non-Gamma patients (Zavascki et al., Brazil, June 2020-May 2021, medium quality)[39]	No data
**Delta**	• Among cases admitted to hospitals, mild cases were relatively lower (P < 0.001) and severe cases higher (*P* < 0.001) in the Delta wave than the first wave (pre-Delta) (Budhiraja et al., India, April 2020-June 2021, medium quality)[29] • Patients with Delta (2.3%) vs. Alpha (2.2%) were more likely to be admitted to hospital within 14 days after a test (aHR] 2.26 [95% CI 1.32–3.89]). Similarly, patients with Delta (5.7%) vs. Alpha (4.2%) were more likely to be admitted to hospital or attend emergency care within 14 days of a test (aHR 1.45 [95% CI 1.08–1.95]) (Twohig et al., UK, March-May 2021, high quality)[70]	• Admission rates were lower in the Delta wave than the pre-Delta wave (23.6% vs. 61.9%) (Khedar et al., India, March 2020-July 2021, medium quality)[43] • No difference was found in the risk of hospitalization among those infected with Delta compared to Alpha (aRR 0.97, 95% CI 0.76–1.23) (Veneti et al., Norway, May-Aug 2021, high quality)[72]
**Combined VOC**	• There was a statistically significant increase in the hospitalization rate for regions in the top 10% percentile of reported VOC cases. Regarding time dynamic effects, the hospitalization rate was ~ 38% higher in high VOC regions (9+ VOC cases) compared to their pre-VOC observation (Mitze and Rode, Germany, Jan-Feb 2021, no appraisal)[77] • Significantly higher proportion of VOC cases were admitted to the hospital compared to non-VOC (Alpha: 11.0%, Beta: 19.3%, Gamma: 20.0% vs. non-VOC: 7.5%, *p* < 0.001). In an adjusted OR in matched multivariable analysis found that VOC cases had higher chance of hospitalization than non-VOC cases (aOR: 1.6-4.2). People aged 20–59 years had 2.3 to 3.0 times greater odds of hospitalization with Alpha compared with non-VOC cases. The highest odds for hospitalization for Beta was 3.5 to 3.6 times higher for age groups 40-79 years compared to non-VOC cases (Funk et al., 7 European countries, Sep 2020-Mar 2021, high quality)[63] • VOC (Alpha, Beta, Gamma) were associated with higher odds of hospitalization (OR 2.25 95% CI 2.10-2.40). These findings were consistent across subgroups (Erman et al., Canada, January-April 2021, medium quality)[32] • Increased rates of hospitalization were seen in VOC infections (all four) relative to non-VOC. Adjusted risk was 59% (95% CI 49-69) higher for hospitalization with VOC (Alpha, Gamma, Beta) than with non-VOC and 120% (95% CI 93-153) higher for hospitalization due to Delta. Increased hospital admission was seen between Delta and other VOC: 55% (95% CI 45-63) (Fisman et al., Canada, Feb-June 2021, medium quality)[33]	No data

#### Impact of VOC on admission to ICU

Twenty-six studies reported on health system impacts related to admission to ICU (see Table 3). Again, Alpha was the VOC most predominantly reported on (*n* = 12 studies), with six studies finding an increase in ICU admission due to Alpha, while six studies reported no change. Fewer studies reported on the other VOC: three studies on Beta (one with increases in ICU admission and two with no change), four studies on Gamma (one reported increases in ICU admission and three no change) and two on Delta (one reported increases in ICU admission and one no change). Five studies reported on combined VOC, with four studies finding an increase in ICU admission compared to non-VOC and one study finding no change. Overall, 50% of studies reported increases in ICU admission due to any VOC compared to non-VOC.

**Table 3:** Summary of findings related to impact of VOC on admission to ICU

VOC	Increased admission to ICU due to VOC	No change in ICU admission due to VOC
**Alpha**	• In both the adjusted and unadjusted analysis, the primary care group had a higher risk of admission to critical care for Alpha patients compared with the non-Alpha patients (adjusted HR: 2.15; 95% CI 1.75 - 2.65). There was no significant interaction between Alpha and sex, ethnic group, or age group (Patone et al., England, Nov 2020-Jan 2021, high quality)[68] • In the Alpha wave, almost half of total ICU patients were admitted (803 out of the total 1680 patients), suggesting greater ICU admission than pre-VOC (Area et al., Spain, March-April 2021, high quality)[62] • The probability of ICU admission was twice as high among patients with Alpha compared to wild-type (OR 2.11, 95 CI% 1.55 − 2.87) (Martinez-Garcia, Spain, Jan-April 2021, medium quality)[50] • ICU admission was higher with Alpha (6.3%) compared to non-Alpha (3.4%) (Pascall et al., Scotland, Nov 2020-Jan 2021, high quality)[67] • Significantly more hospitalized Alpha patients were transferred to ICU (27.7%) compared to 8.6% of non-Alpha patients (Vassallo et al., France, Oct 2020-Apr 2021, medium quality)[38] • Alpha was associated with a 1.8-fold increased risk of ICU admission compared to non-VOC (aRR 95% CI 1.2-2.8) (Veneti et al., Norway, Dec 2020-Jun 2021, high quality)[71]	• There was no significant difference between those admitted to the ICU before Alpha was dominant (23%) compared to after (26 and 35%), *p* = 0.374. For ICU patients, neither the severity score at admission (SAPSII) nor the depth of the respiratory distress seemed to increase with Alpha (Courjon et al., France, Dec 2020-Feb 2021, medium quality)[47] • No difference was found for progressing to critical disease between wild-type and Alpha (Abu-Raddad, Qatar, Jan-May 2021, medium quality)[75] • There was no difference in ICU admission between pre-Alpha and Alpha wave (Cusinato et al., UK, January 2020-March 2021, high quality)[55] • No overall increase in ICU admission was associated Alpha (HR 1.01, (95% CI 0.75-1.37, *p* = 0.94); however, women with Alpha may be at an increased risk of admission to ICU (HR 1.82, 95% CI 1.15-2.90, *p* = 0.011) (Stirrup et al., UK, Nov 2020-Jan 2021, high quality )[69] • There was no change in the proportion of children and young people admitted to critical care between the pre-Alpha (12.9%) and Alpha wave (12.7%) (Swann et al., UK, Jan 2020-Jan 2021, medium quality)[37] • There was no difference between Alpha patients (16%) and non-VOC patients (18%) admitted to the ICU (Whittaker et al., Norway, Dec 2020-Apr 2021, high quality)[73]
**Beta**	• Compared to Alpha, the odds of progressing to critical disease were 1.49-fold (95% CI 1.13-1.97) higher for Beta (Abu-Raddad, Qatar, Jan-May 2021, medium quality)[75] • Beta was associated with a 2.7-fold increased risk of ICU admission compared to non-VOC (aRR 95% CI 1.2-6.5) (Veneti et al., Norway, Dec 2020-Jun 2021, high quality)[71]	• The proportion of patients admitted to the ICU was lower in the Beta wave (35.0%) compared to the pre-Beta wave (48.5%), *p* < 0.001 (Maslo et al., South Africa, June-Dec 2020, medium quality)[44] • No differences for patients admitted to ICU with Beta (7 days) or wild-type (8 days) in terms of days between onset of symptoms (Pascall et al., Scotland, Nov 2020-Jan 2021, high quality)[67]
**Gamma**	• There were more patients admitted to the ICU during the Gamma wave (943) than the pre-Gamma wave (672), particularly among those under 60 years of age and without comorbidities (Nonaka et al., Brazil, May 202-Feb 2021, high quality)[74]	• While there was variation in the age profile of hospitalized patients between Feb 2020-Feb 2021, there was no evidence of an increase in hospitalization in the last period (related to high Gamma) for adults between 18 and 50 years (de Andrade et al., Brazil, Feb 2020-Feb 2021, low quality)[24] • There was no significant difference in the ICU admission risk for maternal patients between 2020 and 2021 (*p* = 0.769) and there was no difference in length of ICU stay (*p* = 0.269) (Takemoto et al., Brazil, Mar 2020-Apr 12th, 2021, medium quality)[52] • There was no difference between ICU admission between Gamma and non-Gamma patients (Zavascki et al., Brazil, June 2020-May 2021, medium quality)[39]
**Delta**	• Over a quarter of pregnant patients diagnosed during high spread of Delta required admission for severe or critical illness, compared to 5.4% before Delta was prominent (Adhikari et al., United States, May-Sept 2021, medium quality)[45]	• Similar amounts of patients were admitted to the ICU in both waves (34.9% in Wave 1 vs. 33.4% in Wave 2 [Delta]), although more patients required oxygen (74.1% vs 63.4%, *p* < 0.001) and invasive ventilation during the Delta wave (10.1% in Wave 2 vs. 8.7% in Wave 1, *p* = 0.002) (Budhiraja et al., India, April 2020-June 2021, medium quality)[29]
**Combined VOC**	• There was an estimated increase of 1.29 (95% CI 0.5-2.1, p < 0.05) additional COVID-19 patients in intensive care per 100,000 population, which is a 42% rise in hospitalization in VOC regions compared to pre-VOC regions (3.08 patients in intensive care per 100,000 population) (Mitze and Rode, Germany, Jan-Feb 2021, no appraisal)[77] • VOC cases were more likely to be admitted to the ICU than non-VOC cases (Alpha: 1.4%, *p* = .002; Beta: 2.3%, *p* = 0.001; Gamma: 2.1%, *p* = 0.005 vs. non-VOC: 0.6%). In an unmatched analysis, VOC were 2.2-3.3 times more likely to be admitted to ICU than non-VOC. ICU admission did not differ for Alpha but increased for Beta (adjusted OR 8; 95% CI 3.7–17.3) only for those aged 40–59 years. For individuals aged 40 or older, there was a 2.9 to 13.9 times higher odds of ICU admission with Gamma than non-VOC (Funk et al., 7 Europe, Sep 2020-Mar 2021, high quality)[63] • VOC (Alpha, Beta, Gamma) were associated with higher rates of ICU admission (OR, 3.31; 95% CI 2.84-3.86) compared to previous strains (Erman et al., Canada, January-April 2021, medium quality)[32] • Increased rates of ICU admission were seen in VOC infections (all four) relative to non-VOC. Adjusted risk was 105% (95% CI 82-134) higher for VOC (Alpha, Gamma, Beta) ICU admission than with non-VOC and 287% (95% CI 198-399) higher for ICU admission due to Delta. Increased ICU admission was seen between Delta and other VOC: 101% (95% CI 79-124) (Fisman et al., Canada, Feb-June 2021, medium quality)[33]	• There were no significant differences between VOC and non-VOC on mean LOS (11.51 vs. 9.56 days), mean critical care LOS (15.25 vs. 18.93 days), or proportion of patients admitted to critical care (0.111 vs. 0.19) (Garvey et al., England, Dec 15th-31st, 2020, high quality) [64]

#### Impact of VOC on mortality

Forty-one studies reported on health system impacts related to risk of mortality (see Table 4). Again, Alpha was the VOC most predominantly reported on (*n* = 23 studies), with eleven studies finding an increase in mortality due to Alpha, four studies reporting mixed findings, and eight studies reporting no change. Five studies on Beta (four reported increases in mortality and one reported mixed findings), six studies on Gamma (all six reported increases in mortality), and three studies on Delta (all three reported increases in mortality) were reported. Four studies reported on combined VOC, with two studies finding an increase in mortality compared to non-VOC and two studies finding no change. Overall, 26/41 studies (63.4%) found an increased risk of mortality due to VOC compared to non-VOC.

**Table 4:** Summary of findings related to impact of VOC on mortality

VOC	Increased mortality due to VOC	Mixed findings in mortality due to VOC	No change in mortality due to VOC
**Alpha**	• An increase of 0.1 in the proportion of Alpha in the population was related with a 15.3% increase in the total number of deaths (Jablońska et al., Europe, Jan-Feb 2021, low quality)[27] • The mortality hazard ratio for people with Alpha compared to those with wild-type was 1.64 (95% CI 1.32 to 2.04). In this community-based, relatively low-risk group, there was a 32 to 104% increased risk of death (Challen et al., UK, Oct 2020-Feb 2021, high quality)[54] • The estimated hazard ratio for Alpha was 1.55 (95% CI 1.39– 1.72), indicating that the risk of mortality in the 28 days following a positive test was 55% (95% CI 39– 72%) higher for Alpha than non-Alpha. Correcting for misclassification and missing SGTF status, this increased to 61% (95% CI 42–82%); however, this was not consistent across age groups, with a greater risk in older age groups (70+) (Davies et al., UK, Nov 2020-Feb 2021, no appraisal )[78] • Alpha was associated with 73% increased risk of death within 28 days compared to non-Alpha cases with the hazard ratio at 1.73 (95% CI 1.41–2.13, *p* < 0.0001) (Grint et al., England, Nov 2020-Jan 2021, high quality)[58] • There is an 18% increase in fatality risk for Alpha compared to non-Alpha with a Case Fatality Rates (CFR) at 1.18 (95% CI 0.40-3.28) (Zhao et al., UK, Sep 2020-Jan 2021, no appraisal)[79] • There was a 33% increase in mortality when considering the effect of Alpha in England (Ackland et al., UK, Sep 21-Nov 5 2020, no appraisal)[80] • The Alpha wave had 39.8% mortality, although the proportion of death in people over 80 was lower: 67.0% compared to 70.9% in across the whole pandemic (Area et al., Spain, March-April 2021, high quality)[62] • There was a marked increase in mortality between pre-Alpha and Alpha wave. The adjusted mortality was 59% (9%5 CI 39–82) higher in high dependency unit and 88% (95% CI 62–118) higher in ICU for the Alpha wave (Dennis et al., UK, March 2020-January 2021, high quality)[56] • VOC-infected patients (primarily Alpha) exhibited higher 30-day risks of death (aOR 1.67, 95% CI 1.13-2.48] in Alberta and aOR 1.52, 95% CI 1.27-1.81] in Ontario) than non-VOC patients (McAlister et al., Canada, March 2020-March 2021, medium quality)[35] • Alpha was associated with a higher risk of death within 28 days than wild-type variants (aHR: 1.59, 95% CI1.44-1.74)) (Nyberg et al., England, Nov 2020-Jan 2021, high quality)[61] • Significantly more hospitalized Alpha patients died (15.4%), compared to 12.9% of non-Alpha patients (Vassallo et al., France, Oct 2020-Apr 2021, medium quality)[38]	• There was an increase in 28-day mortality risk for Alpha compared to non-Alpha patients in both the adjusted and unadjusted model (Adjusted HR: 1.65, 95% CI 1.36-2.01). In the critical care cohort, after adjusting for confounders, critical care mortality did not differ significantly between Alpha and non-VOC Alpha groups (adjusted HR: 0.93, 95% CI 0.76-1.15). Neither cohort had evidence of an interaction between Alpha and ethnic group, age group, or sex (Patone et al., England, Nov 2020-Jan 2021, high quality)[68] • Alpha patients had a slightly higher case-fatality-rate than the non-Alpha patients for younger (e.g. ≤ 70) aged patients, whereas the non-Alpha patients has a higher case-fatality-rate in older ages (Cetin et al., Turkey, April 2020-March 2021, medium quality)[46] • Patients admitted during the Alpha wave had a (crude) mortality rate 25% lower than that of patients admitted during the first wave (IRR 0.75, 95% CI 0.64-0.86). However, in the adjusted analysis, the hazard of death during the Alpha wave was 1.62 times higher (95% CI 1.26-2.08) than during the pre-Alpha wave, considering age, sex, dexamethasone, oxygen requirement, symptoms at admission, and Charlson Comorbidity Index (Cusinato et al., UK, January 2020-March 2021, high quality)[55] • Crude mortality rates were higher during the Alpha wave; however, case fatality rates were lower (Moore et al., Israel, March 2020-Feb 2021, low quality)[26]	• There was no difference in the percentage of patients with and without Alpha who died within 28 days (16% Alpha vs. 17% non-Alpha, *p* = 0.74). In both the unadjusted and adjusted analysis (controlling for hospital, sex, age, comorbidities, and ethnicity), there was no increased risk of mortality or severe disease with Alpha compared to non-Alpha (Frampton et al., UK, Nov-Dec 2020, high quality)[57] • In a matched cohort analysis, there was no evidence of an association between Alpha and non-Alpha on death within 28 days of COVID-19 positive test (OR 0.90, 95% CI 0.57-1.41, *p* = 0.64). After adjusting for confounders (sex, age, ethnicity, residential property classification, week of specimen date and testing Pillar), there was no difference in risk of death among Alpha cases compared to non-Alpha (HR 1.06, 95% CI 0.82-1.38, *p* = 0.65) (Dabrera et al., UK, Oct-Dec 2020, medium quality )[31] • There was no difference found in the death rate between Alpha (0.6%) and non-Alpha individuals (0.9%), *p* = 0.64 (Loconsole et al., Italy, Dec 2020-Mar 2021, medium quality)[48] • There was no increased risk of 28 day mortality after hospitalization between Alpha and wild-type (Martin-Blondel, France, Jan-Feb 2021, medium quality)[34] • There was no difference in mortality between Alpha patients and wild-type (Martinez-Garcia, Spain, Jan-April 2021, medium quality)[50] • Alpha was not associated with increased mortality at 28 days (OR 1.04, 95% CI: 0.67-1.59) (Pascall et al., Scotland, Nov 2020-Jan 2021, high quality)[67] • Alpha was not associated with increased mortality at 28 days overall (HR 1.01, 95% CI 0.79-1.28, *p* = 0.94) (Stirrup et al., UK, Nov 2020-Jan 2021, high quality)[69] • There was no statistically significant difference between Alpha patients (9%) and non-VOC patients (6%) in terms of mortality (Whittaker et al., Norway, Dec 2020-Apr 2021, high quality)[73]
**Beta**	• Compared to Alpha, the odds of COVID-19 death were 1.57-fold (95% CI 1.03-2.43) higher for Beta (Abu-Raddad, Qatar, Jan-May 2021, medium quality )[75] • Adjusting for weekly COVID-19 hospital admissions, there was a 31% increased risk of in-hospital mortality in the Beta wave (aOR 1.31, 95% CI 1.28–1.35) (Jassat et al., South Africa, March 2020-March 2021, high quality)[60] • Beta was highly associated with 60-day mortality in patients admitted to the ICU compared to both Alpha and wild-type (OR 5.67, 95% CI 1.04–30.81) (Louis et al., France, Feb-March 2021, medium quality )[49] • Patients infected with the Beta variant had a higher 28-day in-hospital mortality (32.5%), compared to patients infected with wild-type (22.2%, *p* = 0.1). This excess mortality was confirmed after matching for comorbidities and initial severity (30.6% vs. 19.4%, *p* = 0.04). (Puech et al., France, March 2020-April 2021, medium quality)[36]	• There was no difference in overall mortality between the two waves (36.4% vs. 32.3%), however, ICU mortality was higher in the Beta wave (74.4%) compared to the pre-Beta wave (57.1), *p* = 0.002 (Maslo et al., South Africa, June-Dec 2020, medium quality)[44]	No data
**Gamma**	• There was an 8.2% increase in CFR (15.6% for Gamma from 7.5% wild-type) in maternal deaths out of maternal cases, with the first three months of 2021 accounting for 46.2% of deaths thus far. There was no significant difference in terms of age, type of residence, COVID-19 diagnostic criteria, cardiovascular disease, or diabetes, but the proportion of white women was higher in 2021 (Takemoto et al., Brazil, Mar 2020-Apr 2021, medium quality)[52] • While there were no changes in CFR in children or adolescents, all other groups above 20 years of age had statistically significant increases in CFR when diagnosed in Feb 2021 (Gamma) as opposed to Jan 2021 (non-Gamma). For individuals between 20 and 29 years of age, there was a 3-fold higher risk of death when diagnosed in Feb 2021 compared to Jan 2021 (RR 3.15, 95% CI 1.52-6.53, *p* < 0.01). This risk of death was also higher in other age groups, although to a lesser extent (de Oliveira et al., Brazil, Jan-Feb 2021, low quality)[25] • Each geographical region of Brazil varied in terms of their mortality over the three periods, with the North region being the hardest hit, experiencing a collapse in the provision of healthcare in the first and last periods (Gamma) with high mortality in all age groups (de Andrade et al., Brazil, Feb 2020-Feb 2021, low quality )[24] • The proportion of women who died from COVID-19 increased from 34% in the first wave (non-VOC) to 47% in the second wave (Gamma). There were no significant differences for mortality in males, but the risk of death for men aged 20-39 was more than double in the second wave than the first wave 2.1 (95% CI 1.6-2.8, *p* < 0.0001) and was higher in men aged 40-59 years 1.42 (95% CI1.3-1.6, *p* < 0.0001). Additionally, there was an increase in proportion of deaths for individuals in all age groups (20-59 years) in both sexes (Freitas et al., Brazil, Apr 2020-Jan 2021, low quality)[28] • The CFR was higher across all groups after the emergence of Gamma, with age groups of 20-39 and 40-59 having a higher proportional increase in the second wave than the first wave because of Gamma prevalence. Additionally, people without pre-existing conditions experienced a higher proportional increase in death in the second wave (22%) than the first (13%) (Freitas et al., Brazil, Nov 2020-Feb 2021, medium quality)[30] • 28-day mortality from hospital admission was significantly higher in patients with Gamma than non-Gamma (aHR 3.72; 95% CI 1.19–11.65) (Zavascki et al., Brazil, June 2020-May 2021, medium quality)[39]	No data	No data
**Delta**	• Mortality (25%) was higher among people > 60 years compared to other age group (20-40 years (2%), 40-60 years (14%)) during Delta spread (*p* < .05). Mortality was significantly higher among unvaccinated patients having comorbid conditions than vaccinated patients (*p* < 0.05) (Agrawal et al., India, Dec 2020-June 2021, medium quality)[40] • Mortality during the Delta wave was nearly 40% higher than in pre-Delta wave (10.5% vs. 7.2%, *p* < 0.001), across all age groups, with patients under 45 experiencing the greatest increase (Budhiraja et al., India, April 2020-June 2021, medium quality)[29] • In-hospital deaths were significantly higher in the Delta wave (19.3%), compared to pre-Delta (11.5%) (OR 1.84, 95% CI 1.32-2.55), which did not change significantly with adjustment for age, sex, and comorbidities (Khedar et al., India, March 2020-July 2021, medium quality)[43]	No data	No data
**Combined VOC**	• VOC (Alpha, Beta, Gamma) were associated with higher odds of mortality for both the general COVID-19 population (OR 1.75, 95% CI 1.47-2.09) and hospitalized cases (OR 1.62; 95% CI 1.23-2.15) (Erman et al., Canada, January-April 2021, medium quality)[32] • Increased rates of mortality were seen in VOC infections (all four) relative to non-VOC. Adjusted risk was 61% (95% CI 40-87) higher for VOC (Alpha, Gamma, Beta) mortality than with non-VOC and 137% (95% CI 50-230) higher for mortality due to Delta than non-VOC. Increased mortality was seen between Delta and other VOC: 59% (95% CI 39-84) (Fisman et al., Canada, Feb-June 2021, medium quality)[33]	No data	• There was no difference in mortality between individuals with Alpha or Beta compared to non-VOC (Garvey et al., England, Dec 15-31, 2021, high quality)[64] • There was no increased risk of death for any of the VOC (Alpha, Beta or Gamma) compared to non-VOC (Funk et al., Europe, Sep 2020-Mar 2021, high quality)[63]

### Question B: Adjusting PPE procedures for healthcare workers

One modeling study reported on adjusting Personal Protective Equipment (PPE) procedures. Pham et al. [81] modeled the impact of different interventions on transmission, healthcare worker (HCW) absenteeism, and test positivity as markers of intervention efficiency against Alpha transmission. In the baseline scenario, it was assumed that HCWs were using PPE while in COVID wards when seeing patients but not during breaks or when in other parts of the hospital, assuming 95% of HCWs worked in the same wards over time. While specific PPE used was not defined, PPE efficiency was defined as percentage reduction of droplet transfer. Assuming 90% effective PPE use in COVID wards, they found that extending PPE use to non-COVID wards (all HCWs used PPE with 90% effectiveness when on ward) would prevent 93.7% of all transmissions and would also prevent outbreaks among patients and HCWs. Even if PPE effectiveness was reduced to 70%, findings did not change significantly; however, if it was reduced to 50% or below, screening HCWs every 3 days was more effective than PPE use in all wards. Overall, PPE use in all wards was modeled to be more effective than all other interventions.

One observational study found that the amount of disposable plastic generated by a single RT-PCR diagnostic test and the PPE used by PCR operators was 821.8 g [82]. Given the increased testing with greater spread of COVID-19 due to VOC, the authors argue that there needs to be greater attention paid to biomedical plastic waste to minimize the environmental impact.

### Question C: Adjusting restrictions to and screening staff and visitors (e.g., visitor policy changes, approach to and frequency of screening)

No studies had reported on this outcome as of September 27, 2021.

### Question D: Adjusting service provision based on VOC status (e.g., cohorting patients in hospitals based on the SARS-CoV-2 variants they have)

No studies had reported on this outcome as of September 27, 2021.

### Question E: Adjusting patient accommodations, shared spaces, and common spaces (e.g., improvement to HVAC systems)

One study reported on the presence of SaRS-CoV-2 on regularly-touched environmental surfaces during high Alpha prevalence [82]. In shared spaces/surface contamination, patient bed handles, the nursing station, the reception desk, door handles of doctor’s office, toilet door handles, cell phones, patient toilet sinks, toilet bowls, and patient pillows (defined as high-touch surfaces) were considered as high-risk sources of transmission. Alcohol-based rubs (ethanol 70%) were effective at reducing the presence of SARs-CoV-2 on most surfaces after 15 min where sodium hypochlorite (0.001%) was mostly ineffective [82].

## Discussion

This rapid review sought to identify, appraise, and summarize evidence related to the impact of VOC known as of September 27, 2021, (Alpha, Beta, Gamma, Delta) on health system arrangements. Among the studies that reported on the impact of VOC on hospitalization, trends suggest there is an increase in hospitalization due to VOC. There seems to be less agreement on the impact of VOC on ICU admissions, with only 50% finding an increase in ICU admissions due to VOC. Most studies (63.4%) reporting mortality data found an increased risk of death due to VOC, although health system capacity may influence this. One study reported on the effectiveness of PPE in reducing VOC transmission in the hospital and one study reported on PPE waste and the effectiveness of alcohol-based rubs (ethanol 70%) at reducing the presence of SaRs-CoV-2 on most surfaces after 15 min. No studies reported on screening staff and visitors or adjusting service provisions (e.g., cohorting), which is a significant gap in the literature.

Our search identified 59 studies related to health system arrangements, with almost all reporting on the impact on hospitalization, ICU admissions, and mortality. Due the rapid growth in the literature on VOC and COVID-19 broadly, there is variation in how data is collected, reported, and ultimately summarized. All studies on health system arrangements also came from three primary geographic areas – UK/Europe, Brazil, and France. Thus, the impact of VOC on other health systems around the world are predominantly unreported in the literature to date. Due to variation in study design, conduct, and local epidemiology of COVID-19 and VOC spread, it is difficult to tease apart reasons as to why different studies found variation in the impact of VOC on hospitalization, ICU admission, and mortality rates.

As evident in this rapid review, the findings on the impact of VOC on health system arrangements are quickly changing and emerging. We have identified several specific research gaps that need to be addressed to provide more robust evidence around health system arrangement decisions. In particular, given the lack of evidence this review identified on screening staff and visitors, cohorting patients based on VOC, or adjusting patient accommodations and shared spaces, future research should prioritize these areas to address this gap. Evidence is needed related to best practices for screening staff and visitors in health service organizations and adjusting service provisions. Evidence is also needed to determine whether adjusting patient accommodations and shared spaces in hospital settings is warranted based on the presence of VOC. The generation of evidence from countries that are experiencing significant impacts of VOC and for which there are no current reports should be the focus of future research. Finally, additional research is needed on Beta, Gamma, and Delta to determine whether the risks to health system arrangements are similar for all VOC.

### Limitations

While this rapid review has several strengths, there are limitations that must be acknowledged. First, due to the rapid production of the literature on COVID-19 and VOC, 42% of the studies included in this review were preprints and have thus not yet undergone peer review. Nevertheless, most studies scored medium or high in the quality appraisal, suggesting that the evidence in this area is relatively reliable. Most studies used large health administrative databases as sources of evidence with reliable methods for determining exposures/outcomes. Additionally, our search strategy was limited to articles that specified reporting on one of the recognized VOC (Alpha, Beta, Gamma, and Delta). Given the growing trend that VOC are replacing the wild-type as the dominant strain as well as the continued emergence of other variants of interest, future consideration of expanding the search strategy may be warranted. It is also important to acknowledge the limitation of the epidemiology contact. Due to the variation in testing strategies in countries where studies occurred, the adequacy of case finding in the community and thus denominator completeness may vary, which impacts the ability to assess hospital rates and the impact of VOC on health system impacts and mortality. Finally, some studies reported mixed findings based on adjusted and unadjusted analyses, which must be considered when comparing across studies.

## Conclusions

This rapid review provides synthesized evidence related to the health system impacts of the four SARS-CoV-2 VOC. While the findings should be interpreted with caution as many of the sources identified were preprints, the evidence is trending towards increased risk of severe outcomes including hospitalization and mortality in VOC cases compared to wild type SARS-CoV-2 cases. Currently, there is a lack of pragmatic studies to inform health system capacity expectations and health management practices. Further research is needed to address the gaps identified in this review, including the insufficient or lack of evidence on adjusting PPE procedures for healthcare workers, screening staff and visitors, cohorting patients based on VOC, or adjusting patient accommodations and shared spaces.

## Supplementary Information

**Additional file 1.**

## Data Availability

The datasets used and/or analysed during the current study are available from the corresponding author on reasonable request.

## Abbreviations

Alpha: variant of concern originating in the United Kingdom also known as VUI 202012/01 and VOC 202012/01 formerly known as B.1.1.7
Beta: variant of concern originating in South Africa, also known as 20H/501Y.V2, formerly known as B.1351
Delta: variant of concern originating in India, formerly known as B.1.617.2
Gamma: variant of concern originating in Brazil, also known as B.1.28.1, formerly known as P.1
ARF: acute respiratory failure
CI: confidence interval
CFR: case fatality rate
COG-UK: COVID-19 UK Genetics Consortium
HCW: healthcare worker
HR: hazard ratio
HVAC: heating, ventilation, and air conditioning
ICU: Intensive Care Unit
IQR: interquartile range
LOS: length of stay
OR: odds ratio
PCR: polymerase chain reaction
PPE: personal protective equipment
RR: risk ratio
SGTF: S-gene target failure
UK: United Kingdom
VE: vaccine effectiveness
VOC: variant of concern
WHO: World Health Organization
JBI: Joanna Briggs Institute
