# Supplementary material for: Health system impacts of SARS-CoV − 2 variants of concern: a rapid review
Source: BMC Health Serv Res. 2022 Apr 23;22:544. doi: 10.1186/s12913-022-07847-0 (PMC9034743; doi:10.1186/s12913-022-07847-0)
Supplement: Supplementary file 1 — Additional file 1. [file 12913_2022_7847_MOESM1_ESM.pdf]

Supplementary Material 1: Literature Search Strategy

MEDLINE All (OVID)

COVID-19 search filter: CADTH https://covid.cadth.ca/literature-searching-tools/cadth-covid-19-search-strings/

1	(coronavirus/ or betacoronavirus/ or coronavirus infections/) and (disease outbreaks/ or epidemics/ or pandemics/)
2	(ncov* or 2019ncov or 19ncov or covid19* or covid or sars-cov-2 or sarscov-2 or sarscov2 or severe acute respiratory syndrome coronavirus 2 or severe acute respiratory syndrome corona virus 2).ti,ab,kf,nm,ot,ox,rx,px.
3	((new or novel or "19" or "2019" or wuhan or hubei or china or chinese) adj3 (coronavirus* or corona virus* or betacoronavirus* or CoV or HCoV)).ti,ab,kf,ot.
4	((coronavirus* or corona virus* or betacoronavirus*) adj3 (pandemic* or epidemic* or outbreak* or crisis)).ti,ab,kf,ot.
5	((wuhan or hubei) adj5 pneumonia).ti,ab,kf,ot.
6	or/1-5 [CADTH COVID-19 filter, no date limit]
7	(((uk or united kingdom or england or english or britain or british or kent) adj3 (variant* or voc or vui)) or "b117" or "20i 501yv1" or "variant of concern 202012 01" or "voc 202012 01" or "variant under investigation in december 2020" or "variant under investigation 202012 01" or "vui 202012 01").ti,ab,kw,kf.
8	(((south africa* or sa) adj3 (variant* or voc or vui)) or "b1351" or "501v2" or "501yv2" or "20h 501yv2" or "20c 501yv2").ti,ab,kw,kf.
9	((brazil* adj3 (variant* or voc or vui)) or "p1" or "b11281" or ((mutation* or spike*) adj3 (k417t or e484k or n501y))).ti,ab,kw,kf.
10	((mutation* or spike*) adj3 d614g).ti,ab,kw,kf.
11	((india* adj3 (variant* or voc or vui)) or "b1617*" or "g 452v3" or "voc 21apr" or "vui 21apr" or double mutation or double mutant or double variant or triple mutation or triple mutant or triple variant or ((mutation* or spike*) adj3 (e484q or l452r or p681r))).ti,ab,kw,kf.
12	((alpha or beta or gamma or delta) adj3 variant*).ti,ab,kw,kf.
13	or/7-12
14	6 and 13
	1407 results 2021-09-27

Embase (Elsevier Embase.com)

COVID-19 search filter: CADTH adapted to Embase.com format; line 1 exploded

1	'SARS-related coronavirus'/exp
2	('coronavirinae'/de OR 'betacoronavirus'/de OR 'coronavirus infection'/de) AND ('epidemic'/de OR 'pandemic'/de)

3	(ncov* OR 2019ncov OR 19ncov OR covid19* OR covid OR 'sars-cov-2' OR 'sarscov-2' OR 'sars-cov2' OR sarscov2 OR 'severe acute respiratory syndrome coronavirus 2' OR 'severe acute respiratory syndrome corona virus 2'):ti,ab,kw,de,tt,oa,ok
4	((new OR novel OR '19' OR '2019' OR wuhan OR hubei OR china OR chinese) NEAR/3 (coronavirus* OR 'corona virus*' OR betacoronavirus* OR cov OR hcov)):ti,ab,kw,de,tt,oa,ok
5	((coronavirus* OR 'corona virus*' OR betacoronavirus*) NEAR/3 (pandemic* OR epidemic* OR outbreak* OR crisis)):ti,ab,kw,tt,oa,ok
6	((wuhan OR hubei) NEAR/5 pneumonia):ti,ab,kw,tt,oa,ok
7	#1 OR #2 OR #3 OR #4 OR #5 OR #6
8	(((uk OR 'united kingdom' OR england OR english OR britain OR british OR kent) NEAR/3 (variant* OR voc OR vui)) OR 'b.1.1.7' OR b117 OR '20i 501y.v1' OR 'variant of concern 202012 01' OR 'voc 202012 01' OR 'variant under investigation in december 2020' OR 'variant under investigation 202012 01' OR 'vui 202012 01'):ti,ab,kw
9	((('south africa*' OR sa) NEAR/3 (variant* OR voc OR vui)) OR 'b.1.351' OR b1351 OR '501.v2' OR '501y.v2' OR '20h 501y.v2' OR '20c 501y.v2'):ti,ab,kw
10	((brazil* NEAR/3 (variant* OR voc OR vui)) OR 'p.1' OR p1 OR 'b.1.1.28.1' OR b11281 OR ((mutation* OR spike*) NEAR/3 (k417t OR e484k OR n501y))):ti,ab,kw
11	((mutation* OR spike*) NEAR/3 d614g):ti,ab,kw
12	((india* NEAR/3 (variant* OR voc OR vui)) OR 'b.1.617*' OR b1617* OR 'g 452.v3' OR 'voc 21apr' OR 'vui 21apr' OR 'double mutation' OR 'double mutant' OR 'double variant' OR 'triple mutation' OR 'triple mutant' OR 'triple variant' OR ((mutation* OR spike*) NEAR/3 (e484q OR l452r OR p681r))):ti,ab,kw
13	((alpha OR beta OR gamma OR delta) NEAR/3 variant*):ti,ab,kw
14	#8 OR #9 OR #10 OR #11 OR #12 OR #13
15	#7 AND #14
	1297 results 2021-09-27

Cochrane Library (Wiley)

1	MeSH descriptor: [Coronavirus] this term only
2	MeSH descriptor: [Betacoronavirus] this term only
3	MeSH descriptor: [Coronavirus Infections] this term only
4	{or #1-#3}
5	MeSH descriptor: [Disease Outbreaks] this term only
6	MeSH descriptor: [Epidemics] this term only
7	MeSH descriptor: [Pandemics] this term only
8	{or #5-#7}
9	#4 and #8

10	(ncov* or 2019ncov or 19ncov or covid19* or covid or "sars-cov-2" or "sarscov-2" or sarscov2 or "severe acute respiratory syndrome coronavirus 2" or "severe acute respiratory syndrome corona virus 2"):ti,ab,kw
11	((new or novel or "19" or "2019" or wuhan or hubei or china or chinese) near/3 (coronavirus* or "corona virus*" or betacoronavirus* or cov or hcov)):ti,ab,kw
12	((coronavirus* or "corona virus*" or betacoronavirus*) near/3 (pandemic* or epidemic* or outbreak* or crisis)):ti,ab,kw
13	((wuhan or hubei) near/5 pneumonia):ti,ab,kw
14	{or #9-#13}
15	(variant* or voc or vui or mutation* or spike):ti,ab
16	#14 and #15
	**211 results in CENTRAL 2021-09-27**<br>**3 results in CDSR 2021-09-27**

Epistemonikos LOVE on COVID-19

Basic search of the following terms within the LOVE:
variant* OR voc OR vui OR "ALPHA" OR "20I/501Y.V1" OR "202012/01" OR "B.1.351" OR "501.V2" OR "501Y.V2" OR "20H/501Y.V2" OR "20C/501Y.V2" OR "P.1" OR "B.1.1.28.1" OR "K417T" OR "E484K" OR "N501Y" OR "D614G" OR "B.1.617" OR "B.1.617.1" OR "B.1.617.2" OR "B.1.617.3" OR "G/452.V3" OR "VOC-21APR" OR "VUI-21APR" OR "double mutation" OR "double mutant" OR "double variant" OR "E484Q" OR "L452R" OR "P681R"
3494 results 2021-09-27

medRxiv / bioRxiv

medRxiv and bioRxiv simultaneous search; Title and Abstract search; All words (unless otherwise specified); 50 per page; Best Match; first 50 results of each search query exported; internal duplicates removed using Covidence

Original search (limited October 1, 2020 – May 11, 2021) - 423 results through May 11, 2021

uk variant<br>united kingdom variant<br>england variant<br>english variant<br>britain variant<br>british variant<br>kent variant<br>south africa variant<br>brazil variant<br>variant of concern *(phrase search)*<br>variants of concern *(phrase search)*<br>ALPHA<br>20I/501Y.V1<br>202012/01

B.1.351
501.V2
501Y.V2
20H/501Y.V2
20C/501Y.V2
P.1
B.1.1.28.1
K417T
E484K
N501Y
D614G
india variant
B.1.617
B.1.617.1
B.1.617.2
B.1.617.3
G/452.V3
VOC-21APR
VUI-21APR
double mutation *(phrase search)*
double mutant *(phrase search)*
double variant *(phrase search)*
E484Q
L452R
P681R

Updated search (May 11, 2021 – September 27, 2021) - 465 results through September 27, 2021

alpha variant
beta variant
gamma variant
delta variant
uk variant
united kingdom variant
england variant
english variant
britain variant
british variant
kent variant
south africa variant
brazil variant
india variant
variant of concern *(phrase search)*
variants of concern *(phrase search)*

Search for any (all at once):
Group 1
B.1.1.7

20I/501Y.V1
202012/01
B.1.351
501.V2
501Y.V2
20H/501Y.V2
20C/501Y.V2
P.1
B.1.1.28.1
K417T
E484K
N501Y
D614G

Group 2
B.1.617
B.1.617.1
B.1.617.2
B.1.617.3
G/452.V3
VOC-21APR
VUI-21APR
E484Q
L452R
P681R

Supplementary Material 2: Summary Tables of Included Studies

Author, date	Date of data collection	Source	Objective	Setting	Sample size	Outcome measures	Variant	Country	Main finding
Abu-Raddad, 2021 [74]	Jan 18-May 31, 2021	Clinical Infectious Diseases	To conduct a comparative assessment of the severity, criticality, and fatality of Alpha & Beta	Community and hospital	NR	Acute-care hospitalization, ICU hospitalization, and mortality	Alpha, Beta	Qatar	Compared to Alpha variant, odds of progressing to severe disease were 1.24-fold (95% CI: 1.11-1.39) higher for Beta, odds of progressing to critical disease were 1.49-fold (95% CI: 1.13-1.97) higher, and odds of COVID-19 death were 1.57-fold (95% CI: 1.03-2.43) higher.
Ackland, 2021 [79]	Apr 22nd, 2021	medRxiv	To track the statistical CFR in the second wave of the UK outbreak, and to understand its variations over time.	Community	NR	Estimates of CFR and their variations over time	Alpha	UK	The relationship between cases and deaths, even when controlling for age, is not static through the second wave of coronavirus in England. The rapid growth in CFR in December can be understood in part in terms of a more deadly new variant Alpha, while a decline in January correlates with vaccine roll-out.
Adhikari, 2021 [44]	May 17, 2020 - Sept 4, 2021	American Journal of Obstetrics & Gynecology	To report the trends of illness severity among obstetrical patients with COVID-19, on a background of Delta variant predominance and describe COVID-19 vaccinations in this cohort at Parkland Hospital.	Hospital	1515 pregnant patients diagnosed with COVID-19	Severe or critical COVID-19 illness in pregnancy	Delta	USA	Increased morbidity was observed in pregnancy with COVID-19 during the recent surge associated with the Delta variant, particularly where vaccine acceptance is low.
Agrawal, 2021 [39]	Dec 15, 2020-June 15, 2021	European Journal of Molecular & Clinical Medicine	To analyze and establish a correlation of demographic features and vaccination status	Hospital	478 sequenced samples among which 100 samples were	Mortality	Alpha, Delta	India	Vaccination has an important protective role in decreasing mortality, especially against the Delta variant for old age

Author, date	Date of data collection	Source	Objective	Setting	Sample size	Outcome measures	Variant	Country	Main finding
			with clinical outcome of VOC's.		reported as VOC				patients with associated co-morbidities.
AlQahtani, 2021 [50]	Dec 9, 2020 - July 17, 2021	Research Square	To present an analysis of COVID-19 related outcomes from Bahrain, where four vaccines were deployed, and provide a granular description of the effectiveness of the four vaccines, disease burden in unvaccinated versus vaccinated individuals over time, and the risk of four outcomes (infections, hospitalizations, ICU admissions and deaths) due to breakthrough infections among vaccinated individuals	Community	1,003,960	Histories of vaccinated vs. unvaccinated cases in terms of infections, hospitalizations, ICU admissions and deaths	Delta	Bahrain	All four vaccines decreased the risk of SARS-CoV-2 infections, hospitalizations, ICU admissions and deaths among vaccine recipients when compared to unvaccinated individuals. However, individuals who received the Sinopharm experienced a higher rate of infection, hospitalization, ICU admission and deaths compared to those who received the Pfizer/BioNtech vaccine.
Area, 2021 [61]	March 1st 2020- April 1, 2021	International Journal of Environmental Research and Public Health	To perform a descriptive statistical analysis of real data after one year of the pandemic in Galicia (Spain), by considering newly infected individuals, admissions to hospitals, admissions to intensive care units and deceased individuals.	Community	NR	Newly infected individuals, hospital and ICU admission, mortality	Alpha	Spain	There were more ICU admissions and mortality in the Alpha Wave compared to previous waves.
Bager, 2021 [52]	Jan 1- March 24, 2021	The Lancet	To assess the risk of COVID-19 hospitalisation in individuals infected with Alpha compared with those with other SARS-CoV-2 lineages	Community & hospital	35,887 test-positive individuals were identified, 11.6% with Alpha	Hospital admission within 14 days after a positive SARS-CoV-2 PCR test or 48hr before a positive test	Alpha	Denmark	Infection with Alpha was associated with an increased risk of hospitalisation compared with individuals infected with other lineages of SARS-CoV-2 virus.
Budhiraja, 2021 [28]	April 2020 - June 2021	medRxiv	To investigate the differentials in the characteristics of the cases admitted in the second wave versus	Hospital	N=14398 cases in first wave (2020) N=5454 cases in	Demographic profile, Clinical features (total cases; ICU/HDU admission; readmission; duration of	Delta	India	No significant demographic differences in the cases in these two waves, indicates the role of other factors such as

Author, date	Date of data collection	Source	Objective	Setting	Sample size	Outcome measures	Variant	Country	Main finding
			those admitted in the first wave.		second wave (2021)	hospitalization; oxygen therapy; drug therapy), Mortality			Delta variant and late admissions in higher severity and more deaths.
Cetin, 2021 [45]	April 2020 - March 2021	Acta Microbiologica et Immunologica Hungarica	To collect and to analyze data related to the infection caused by different variants	Hospital	3111 COVID-19 patients, 596 in-patients	Hospital stay time, case-fatality-rate, mortality	Alpha	Turkey	The Alpha variant increases the risk for hospitalization, increases the CFR for lower age group, and decreases the CFR for the upper age group
Challen, 2021 [53]	Oct 1st, 2021-Feb 12th, 2021	BMJ	To establish whether there is any change in mortality from infection with Alpha compared with wild-type	Community	54,906 matched cohort pairs (on age, sex, and ethnicity) of participants who tested positive for SARS-CoV-2	Death within 28 days of the first positive SARS-CoV-2 test result	Alpha	UK	Probability that the risk of mortality is increased by infection with Alpha is high; infection with Alpha has the potential to cause substantial additional mortality compared to wild-type
Courjon, 2021 [46]	Dec 2020-Feb 2021	Nature	To analyze modification in clinical profile and outcome traits when Alpha was emerging	Hospital	ED (n=1247) & Infectious disease ward or ICU (n=232)	Profile of COVID-19 patients admitted in ED; Comparison of hospitalized patients	Alpha	France	There was no significant difference on time from first symptoms to ED admission, severity, need for immediate ICU management, ICU admission, or severity score on admission between Alpha and wild-type
Cusinato, 2021 [54]	Jan 1, 2020 and March 31, 2021	Infection	To assess whether mortality of patients admitted for COVID-19 treatment was different in the second UK epidemic wave of COVID-19 compared to the first wave accounting for improvements in the standard of care available and differences in the distribution of risk factors between the two waves	Hospital	3 ,376 covid-19 positive cases, of these, 2,701 were patients admitted to covid-19 wards for treatment (32.7% (884 of 2701) in the first wave and 67.3% (1817 of 2701) in the second wave)	In-hospital covid-19-associated mortality and ICU admission	Alpha	UK	Although crude rates would indicate a lower in-hospital mortality during the second wave; accounting for differences in the distribution of protective and risk factors, suggests a higher risk of death during the second epidemic wave compared to the first.

Author, date	Date of data collection	Source	Objective	Setting	Sample size	Outcome measures	Variant	Country	Main finding
Dabrera, 2021 [30]	Oct 2020-Jan 2021	SSRN	To assess whether infection with Alpha were associated with more severe clinical outcomes compared to wild-type infection	Community	63,609 genomically sequenced COVID-19 cases	Risk in hospitalization and risk of mortality within 28 days of test	Alpha	UK	There was a 34% increased risk in hospitalization associated with Alpha compared to wild-type cases, however, no significant difference in the risk of mortality was found after adjusting for confounders.
Davies, 2021 [77]	Sep 1st, 2020-Feb 14th, 2021	Nature	To describe association between SGTF and hazard of death/disease severity	Community	2,245,263 individuals with a positive test, 51.1% of which had a conclusive SGTF reading and, of these, 58.8% had SGTF (suggesting Alpha)	COVID-19 death occurring within 28 days of an individual's first positive COVID test	Alpha	UK	The hazard of death in the 28 days following a positive test is 55% (39–72%) higher for Alpha than for wild-type. Correcting for misclassification of SGTF and missing data on SGTF status, this increases to 61% (42–82%). Alpha is not only more transmissible than pre-existing SARS-CoV-2 variants but may also cause higher mortality.
de Andrade et al., 2021 [23]	Feb 16th 2020 - Feb 20, 2021	medRxiv	To compare during the first year of the pandemic the age profile of patients hospitalized by COVID-19, as well as hospital mortality and use of ICUs, by age group	Hospital	720,36 completed records of patients hospitalized by COVID-19	Hospital mortality and use of ICUs	Gamma	Brazil	Each geographical region of Brazil varied in terms of their mortality over the year, with the North region being the hardest hit, experiencing a collapse in the provision of healthcare in the first (initial introduction of wild-type) and last period (associated with Gamma) with high mortality in all age groups.
de Oliveira et al., 2021 [24]	Sep 1st, 2020-Mar 17th, 2021	medRxiv	To assess recent trends in mortality data among different age-grouped populations in Brazil	Community	553,518 individuals infected with SARS- CoV-2 in Parana	CFRs	Gamma	Brazil	There was an 80-215% increased risk of mortality for adults in different age categories between 20-59 years

Author, date	Date of data collection	Source	Objective	Setting	Sample size	Outcome measures	Variant	Country	Main finding
					between Sep 2020 and Mar 2021				between Feb 2020 and Jan 2021, when Gamma was prominent.
Dennis, 2021 [55]	Mar 1, 2020 - Jan 31, 2021	Critical Care Medicine	To determine whether the previously described trend of improving mortality in people with COVID-19 in critical care during the first wave was maintained, plateaued, or reversed during the second wave in United Kingdom, when Alpha became the dominant strain	All English hospital trusts	49,862	In-hospital 28-day mortality by calendar month of admission	Alpha	UK	There was a marked deterioration in outcomes for patients admitted to critical care at the peak of the second wave of COVID-19 in United Kingdom, compared with the post-first-wave period.
Di Domenico, 2021[75]	Jan 7-8 2021	Eurosurveillance	To assess the impact of implemented measures on two COVID strains (i.e., Alpha and wild-type) through modeling	Community	N/A	Estimated number cases of wild-type and Alpha based on social distancing measures using data from a large-scale genome sequencing initiative conducted in France	Alpha	France	Social distancing implemented in Jan 2021 would bring down the R of historical strain, however VOC would continue to increase. School holidays also slowed down dynamics. Accelerating vaccinations will help but won't be sufficient to stop the spread of the VOC, even with optimistic vaccination rates.
Erman, 2021 [31]	Jan 1 - Apr 9, 2021	medRxiv	To estimate healthcare resource use and health outcomes attributable to VOCs introduced to Ontario between January 1 and April 9, 2021, relative to the previously circulating wild-type strain	Hospital	77,200 subjects	VOC-attributable odds of hospitalisation, ICU admission and death among all infected subjects in the general COVID-19 population	Alpha, Beta, Gamma	Canada	This study demonstrates worse outcomes for patients with VOC. Health systems may face increased demand for healthcare resources as VOC predominate worldwide in view of low global vaccination coverage.
Fisman, 2021 [32]	Feb 7 - June 22, 2021	medRxiv	To evaluate the virulence of VOC compared to non-VOC SARS-CoV-2 infections	Community and hospital	212,332	Risk of hospitalization, intensive care unit (ICU) admission, and death	Alpha, Beta, Gamma, Delta	Canada	The progressive increase in transmissibility and virulence of VOC will result in a significantly larger, and more deadly, pandemic than would

Author, date	Date of data collection	Source	Objective	Setting	Sample size	Outcome measures	Variant	Country	Main finding
									have occurred in the absence of VOC emergence. This is demonstrated by increased hospitalizations and deaths.
Frampton, 2021 [56]	Nov 9th Dec 20th, 2020	Lancet	To describe emergence of Alpha in two North Central London hospitals including comparing virological characteristics and clinical outcomes	Hospital	Of 496 patients with samples positive for SARS-CoV-2 on PCR, 341 had samples that could be sequenced. 58% had Alpha	Severe disease (defined as point 6 or higher on the WHO ordinal scale within 14 days of symptoms or positive test) and death within 28 days of a positive test	Alpha	UK	While length of stay, risk of hospitalization within 14 days of a test, and time to hospital admission from symptom onset were similar, patients with Alpha were younger, had fewer comorbidities and more likely to be from an ethnic minority. There was no increased risk of mortality or severe disease with Alpha compared to wild-type.
Freitas, 2021 [27]	Apr 2020-Mar 1st, 2021	SciELO - Preprints	To describe and identify potential changes in the mortality profile associated temporally with the emergence of the Gamma strain in the state of Amazonas	Community	In the first wave of the COVID-19 pandemic in Amazonas, 46,342 cases were recorded and, in the second wave, 61,273 cases	Demographic data, clinical data, comorbidities, hospitalization, ICU admission, need ventilatory support, hospital outcomes	Gamma	Brazil	Findings suggest that simultaneously with the emergence of Gamma in the state of Amazonas, there was an increase in the proportion of deaths in women and in the populations between 20 and 59 years of both sexes. There were also relative increases in the different age groups and genders in the mortality, lethality, and hospital lethality rates.
Freitas, 2021 [29]	Nov 2020-Feb 2021	medRxiv	To provide an epidemiological analysis describing and comparing the severity and mortality profile of COVID-19 cases considering two periods	Community	230,986 cases of COVID-19 were confirmed in the first wave and 150,942 cases in the second wave	Demographic data, presence of pre-existing conditions, onset of symptoms, hospitalization, and hospital outcome, as well as data on the	Gamma	Brazil	There was an increase in the proportion of young people and people without previous illnesses among severe cases and deaths after the identification of the local transmission of

Author, date	Date of data collection	Source	Objective	Setting	Sample size	Outcome measures	Variant	Country	Main finding
			before and after the emergence of Gamma			occupation of ward beds and ICU beds			variant Gamma. There was also an increase in the proportion of severe cases and in the CFR, in almost all subgroups analyzed. This increase was heterogeneous in different age groups and sex.
Funk, 2021 [62]	Weeks 38/2020-10/2021	Euro Surveillance	To analyze COVID-19 cases infected with any of the three severe VOC (Alpha, Beta, Gamma) and compare them with wild-type with a focus on disease severity	Community	23,343 had information on SARS-CoV-2 variants, of which 19,995 were VOC and 3,348 wild-type cases included	Information on sex, age, clinical symptoms, pre-existing conditions, hospital and ICU admission and outcome (i.e., survived or died).	Alpha, Beta, Gamma	Cyprus, Estonia, Finland, Ireland, Italy, Luxembourg, and Portugal	An increased risk for hospitalizations and ICU admission was associated with all three VOC.
Garvey, 2021 [63]	Dec 15th-31st, 2020	Journal of Infection	To report, observations and outcomes of Alpha infected patients admitted to UHB during Dec 2020	Hospital	152 - 79 were Alpha and 1 was Beta	Clinical outcomes: hospital length of stay, critical care admission, critical care length of stay, treatment given for COVID-19, oxygen and ventilatory support, & death	Alpha, Beta	UK	Alpha was associated with younger age; an increased proportion of patients being admitted to critical care for longer periods, but the association wasn't statistically significant. Whilst numbers of patients were relatively low, no increase in mortality was observed.
Graham, 2021 [64]	Sep 8th-Dec 31st, 2020	Lancet Public Health	To examine the association between the regional proportion of Alpha and reported symptoms, disease course, rates of reinfection, and transmissibility	Community	36,920 COVID-19 positive users of the COVID symptom app. Surveillance data from the (COG-UK) and a SGTF correlate in community testing data.	Regional proportion of Alpha and symptoms, disease course, rates of reinfection and transmissibility.	Alpha	UK	No evidence of changes in reported symptoms, disease severity and disease duration associated with Alpha.
Grint, 2021 [57]	Nov 16th, 2020-Jan 11th, 2021	Clinical Infectious Diseases	To describe the severity of the Alpha variant in terms of the pathway of disease from testing	Community	185,234 people tested positive for SARS-CoV-2 in the community	All-cause mortality based on relative hazard of death ratio and absolute risk of death by 28 days	Alpha	England	Alpha was associated with two-thirds higher case fatality than wild-type in this unvaccinated population.

Author, date	Date of data collection	Source	Objective	Setting	Sample size	Outcome measures	Variant	Country	Main finding
			positive to hospital admission and death		(Alpha = 93 153; wild-type = 92 081)				
Haas, 2021 [58]	Jan 24th- Mar 6th, 2021	Lancet	To provide nationwide estimates of the effectiveness of two doses of Pfizer against SARS-CoV-2 outcomes, and document the first nationwide public-health impact following the widespread introduction of the vaccine at the population level	Community	There were 202 684 SARS-CoV-2 infections in Israel, of which 93.9% was Alpha. There were 6,040 hospitalizations, 3,470 severe and critical hospitalizations, and 754 deaths among persons aged >15 years	Range of SARS-CoV-2 outcomes, including all SARS-CoV-2 infections (symptomatic and asymptomatic), hospitalizations (severe and critical) and deaths	Alpha	Israel	Two doses of Pfizer >7 days after admission were highly effective in preventing hospitalizations, severe and critical hospitalizations, and deaths at a time when Alpha was the dominant strain.
Havers, 2021 [41]	Jan 1– June 30, 2021	medRxiv	To examine characteristics associated with vaccination breakthrough	hospital	4,732 cases with hospitalization associated primarily with COVID-19 illness, including 4,440 unvaccinated and 292 fully vaccinated cases	Descriptive characteristics, vaccination status, hospitalization	Delta	USA	Population-based hospitalization rates show that unvaccinated adults aged ≥18 years are 17 times more likely to be hospitalized compared with vaccinated adults. Rates are far higher in unvaccinated persons in all adult age groups, including during a period when Delta was the predominant strain.
Jabłońska, 2021 [26]	June 2020 - Feb 25th, 2021	medRxiv	To detect potential association between COVID-19 mortality and proportion of Alpha through the second wave of the pandemic in Europe with the use of multivariate regression models	Community	A dataset of 3971 SARS-CoV-2 virus strains identified between Dec 2019 and Mar 2021	COVID-19 deaths during the second wave	Alpha and 11 other variants	38 European countries	Findings suggest that the development and spread of Alpha had a significant impact on the mortality during the second wave of COVID-19 pandemic in Europe.
Jassat, 2021 [59]	Mar 5, 2020 - Mar 27, 2021	Lancet Global Health	The aim is to compare in-hospital mortality and other patient characteristics between the first and second	Hospital	1,545,431 SARS-CoV-2 cases and 227,932 COVID-19	Hospital admissions, deaths	Beta	South Africa	In South Africa, the second wave was associated with higher incidence of COVID-19, more rapid increase in

Author, date	Date of data collectio n	Source	Objective	Setting	Sample size	Outcome measures	Variant	Country	Main finding
			waves, with reference to Beta		hospital admissions				admissions to hospital, and increased in-hospital mortality. This is felt to be multifactorial including system pressure, however Beta variant felt to be significant contributor to increasing mortality rates.
Khedar, 2021 [42]	Mar 2020 - July 2021	medRxiv	To compare demographic, clinical and laboratory features and outcomes in the second Covid-19 wave in India (driven by Delta) with the previous wave	Hospital	First wave: 7476 tested, 1395 COVID-positive, 863 hospitalized; Second wave: 8680 tested, 1641 COVID-positive, 388 hospitalized	Descriptive characteristics, hospitalizations, in-hospital interventions, ICU admissions, deaths	Delta	India	Patients admitted during the second wave had greater length of stay in intensive care unit, oxygen requirement, non-invasive and invasive ventilatory support. The in-hospital mortality in the second wave was double of the first.
Loconsole, 2021 [47]	Dec 22nd, 2020-Mar 9th, 2021	Environmental Research and Public Health	To evaluate the spread of Alpha in southern Italy from Dec 2020-Mar 2021	Community	3,075	Positive SGTF detection, symptomology (i.e., hospitalization)	Alpha	Italy	Alpha cases were more likely to be symptomatic and to result in hospitalization.
Louis, 2021 [48]	Feb 3rd - March 16th 2021	Anaesthesia Critical Care & Pain Medicine	To report the preliminary observational data of consecutive COVID-19 critically ill patients infected by Beta	Hospital	104	Demographics, ICU scores, comorbidities, biological data, chest computed tomodensitometry and clinical outcomes of patients were compared across variant strains (V1, V2 and wild-type)	Beta	France	Beta was associated with high short-term mortality and could be more pathogenic than Alpha.
Martin-Blondel, 2021 [33]	Jan 1st - Feb 28, 2021	SSRN	To assess the effect of Alpha on severity in comparison to other COVID-19 lineages	Hospital	650 with alpha, 650 with other lineages	Primary outcome: proportion of participants with a severe form of COVID-19 occurring up to day 29<br>30 after the date of hospitalization (severity as defined by WHO clinical progression scale); Secondary outcomes: mortality,	Alpha	France	Alpha is associated with increased risk of hospitalization in younger patients and severity in patients that are less comorbid and may lead to increased mortality.

Author, date	Date of data collection	Source	Objective	Setting	Sample size	Outcome measures	Variant	Country	Main finding
						admission to ICU, invasive ventilation and other clinical statistics of interest			
Martinez-Garcia, 2021 [49]	Jan 2nd t-Apr 30th, 2021	Microorganisms	To evaluate how Alpha has replaced the other ones circulating in our geographic area & to study its transmissibility and the associated burden of disease	Hospital	27,633	Prevalence of Alpha and proportion in comparison to other strains; Burden of disease in hospitalized patients	Alpha	Spain	Alpha is associated with increased hospitalization and more severe infection, and individuals infected with this variant have a higher need for ICU and IRCU admission.
Maslo, 2021 [43]	June 14 - Dec 19, 2020	medRxiv	To assess characteristics, severity of illness and mortality in patients hospitalised during the second wave and compared this with those hospitalised in the first wave	Hospital	303 patients in the first wave, 357 in the second	Patient characteristics, therapy, highest level of care, ventilation requirements, length of stay, mortality and laboratory data	Beta	South Africa	Beta is possibly more virulent, with increased mortality and higher ICU mortality.
McAlister, 2021 [34]	Mar 1, 2020 - Mar 31, 2021	medRxiv	To examine 30-day outcomes in Canadians infected with SARS-CoV-2 in the first year of the pandemic and to compare event rates in those with VOC versus wild-type infection	Community	104,232 (in wave 3)	All cause hospitalization or death within 30 days of positive test	Alpha, Beta, Gamma, Delta	Canada	There was a shift towards younger age groups getting infected in the third wave, resulting in more hospitalizations but shorter length of stay. On an individual basis, VOC infection was associated with higher risk of hospitalization or death than wild-type.
Mitze and Rode, 2021 [76]	Dec 15th, 2020-Feb 4th, 2021	MedRxiv	To provide estimates of the epidemiological trends associated with the reporting of Alpha and wild-type for two key indicators: i) the 7-day incidence rate, and ii) the hospitalization rate	Community & hospital (linked data)	Data on daily SARS-CoV-2 infection data for each of the health regions from the COVID-19 dashboard of the Robert Koch Institute. The number of hospitalized patients in ICU was taken from	Comparing the development in epidemiological outcome variables of two groups (wild-type and Alpha). Outcomes of interest were i) the 7-day incidence rate and ii) the hospitalization rate.	Pooled information on VOC	Germany	There was a significant increase in the hospitalization rate in regions in the top 10% percentile of reported VOC cases with an estimated increase of 1.29 [CI: 0.5, 2.1] additional COVID-19 patients in intensive care per 100,000 population.

Author, date	Date of data collection	Source	Objective	Setting	Sample size	Outcome measures	Variant	Country	Main finding
					the INFAS Corona database.				
Moore, 2021 [25]	Mar 1, 2020 - Feb 28, 2021	medRxiv	To analyze the Israeli data on COVID-19 to determine whether the Alpha variant produced a higher fatality rate	Community	NR	Mortality and case fatality rates on a weekly basis	Alpha	Israel	Although Alpha variant appears more transmissible, findings suggest that it is not more deadly once a patient is infected.
Nonaka, 2021 [73]	May 2020 - February 2021	International Journal of Infectious Diseases	To evaluate changes in the characteristics of patients with COVID19 after the emergence of the Gamma, by comparing the clinical, demographic, and laboratory profiles of patients hospitalized during the first (May to July 2020) and second (December 2020 to February 2021) pandemic waves	Hospital	4,164	Confirmed COVID-19 hospitalizations, ICU admissions, clinical (comorbidities) and demographic profiles	Gamma	Brazil	An increased proportion of younger adults without comorbidities with severe disease during the second COVID-19 wave, shortly after the confirmation of local Gamma circulation.
Nyberg, 2021 [60]	Nov 23, 2020- Jan31, 2021	BMJ Open	To evaluate the relation between diagnosis of COVID-19 with Alpha and the risk of hospital admission compared with diagnosis with wild-type	Community based testing, individually linked with hospital admission data	839,278 with laboratory confirmed COVID-19 of whom 36,233 admitted to hospital within 14 days	Hospital admission between 1 and 14 days after first positive test	Alpha	England	Risk of hospital admission is higher for people infected with the Alpha variant compared with wild-type, likely reflecting a more severe disease, and this higher severity may be specific to adults over 30 years old.
Ong, 2021 [65]	Dec 20, 2020 - May 12, 2021	Clinical Infectious Diseases	To compare outcomes of patients infected with Alpha, Beta, and Delta with those with wild-type, with the aim of improving our understanding of the relationship between viral variants, disease severity and viral shedding kinetics	Hospital	2,930 confirmed cases; 829 VOC	Primary outcomes development of pneumonia, oxygen requirement, ICU admission, and death. Secondary outcomes were comparison of viral PCR Ct values and duration of viral shedding between VOCs.	Alpha, Beta, Delta	Singapore	An association was found between Delta and increased disease severity.

Author, date	Date of data collection	Source	Objective	Setting	Sample size	Outcome measures	Variant	Country	Main finding
Pascall, 2021 [66]	Nov 1, 2020 - Jan 30, 2021	MedRxiv	To quantify the clinical features and rate of spread of Alpha infections in Scotland in a comprehensive national dataset	Hospital and community cases	1,475 (364 with Alpha); 59% of sample hospitalized and 41% community	Clinical outcomes by 28 days using a 4-point scale of maximum severity: 1. no respiratory support, 2. oxygen, 3. ventilation and 4. death	Alpha	Scotland	Alpha variant associated with more severe clinical disease.
Patone, 2021 [67]	Nov 1st, 2020- Jan 27th, 2021	The Lancet	To estimate the risk of critical care admission, mortality in critically ill patients, and overall mortality associated with Alpha compared with the wild-type	Community & hospital (linked data)	The ‘primary care cohort’ was patients in primary care with a positive community COVID-19 test reported between 1 November 2020 and 26 January 2021. The first cohort included 198,420 patients. Of these, 117926 had Alpha.<br><br>The ‘critical care cohort’ was patients admitted for critical care with a positive community COVID-19 test reported between 1 November 2020 and 27 January 2021. 4272 had SGTF status and were included. Of these, 2685 had Alpha	For the primary care cohort, critical care and 28-day mortality. For the critical care cohort, duration of organ support (respiratory, cardiovascular, renal, neurological and liver) in critical care, duration of critical care and mortality at the end of critical care.	Alpha	England	Patients with Alpha were at increased risk of ICU admission and 28-day mortality compared with patients with non-Alpha. For patients receiving critical care, mortality appeared to be independent of virus strain.
Pham, 2021 [80]	Feb-Aug 2020	BMC Medicine	To explore the effectiveness of different infection prevention strategies for	Hospital	N/A	The effective reproduction number for patients and HCWs. Also measured HCW	Alpha	Netherlands	In response to the emergence of more transmissible VOC, universal PPE use in all

Author, date	Date of data collection	Source	Objective	Setting	Sample size	Outcome measures	Variant	Country	Main finding
			HCWs in hospitals in the absence of vaccination using an agent-based model of nosocomial SARS-CoV-2 transmission			absenteeism and numbers of nosocomial infections			hospital wards is the most effective in preventing nosocomial transmission and is the most effective intervention to reduce the reproduction number and absenteeism. Regular screening and contact tracing of HCWs are also effective interventions, but critically depend on the sensitivity of the diagnostic test used.
Puech, 2021 [35]	Mar 1 2020 - Apr 18 2021	Research Square	To compare the prognosis of patients with ARF due to Beta to that of patients with ARF due to wild type	Three hospital ICUs (in the Reunion Island University Hospital system)	218 nasopharyngeal or respiratory samples	The primary outcome was 28-day in-hospital mortality. The secondary outcomes were the occurrence of pulmonary embolism, the occurrence of hospital acquired pneumonia, the need for venovenous extracorporeal membrane oxygenation support, and in-ICU length of stay.	Beta	France	Mortality was higher in patients with ARF infected with Beta than in patients infected with wildtype. Given that there were optimal management conditions, the excess mortality observed in the study cohort is likely explained by the increased virulence of the Beta variant relative to the original strain.
Seif, 2021 [81]	April 23, 2021	Environmental Research	To determine the presence of the virus on regularly-touched environmental surfaces, as well as the persistence of the virus to two common types of disinfectants, and finally, to estimate the plastic waste produced by the real-time PCR test and its operators in the hospital	Hospital	76 samples from environmental surfaces that were obtained from different hospital wards before disinfection and cleaning	Presence of SARS-CoV-2 on high-touch environmental surfaces; Persistence of SARS-CoV-2 on different inanimate surfaces after using disinfectants; Spread of the disease was measured in both the wards with confirmed cases as well as the non-infectious wards; amount of disposable plastic waste generated by diagnostic tests and PPE	Alpha, Beta	Iran	Beta can survive on inanimate surfaces after disinfection with alcohol-based hand runs (ethanol 70%) up to 5 minutes but in most cases, not more than 15 minutes. An increased level in plastic waste was inevitable.

Author, date	Date of data collection	Source	Objective	Setting	Sample size	Outcome measures	Variant	Country	Main finding
Snell, 2021 [40]	Mar 13th, 2020 and Feb 17th, 2021	medRxiv	To compare admission characteristics of hospitalized cases during the two dominant waves of infection for local healthcare planning	Hospital	2341 total; 838 in wave 1 and 1503 in wave two	Comparison of demographic, physiological and laboratory parameters of hospitalized SARS-CoV-2 positive cases during Wave 1/wild-type and Wave2/Alpha extracted from hospital electronic health record	Alpha	UK	While there was double the admissions in Wave 2 (Alpha), patients with Alpha were similar in age and ethnicity compared to wild-type. Alpha patients were less likely to be frail but more likely to be obese hypoxic on admission, the main indicator of severe disease, than patients with wild-type.
Stirrup, 2021 [68]	Nov 16, 2020 - Jan 10, 2021	BMJ Open Respiratory Research	To estimate the overall effect of Alpha on mortality and ICU admission and to evaluate whether the impact of the variant differed according to patient characteristics	Hospital	Sequences were obtained from 2341 inpatients and analysis of clinical outcomes was carried out in 2147 inpatients with all data available.	Mortality and Intensive Therapy Unit admission (both in patients admitted with COVID-19 and hospital onset COVID-19 infections)	Alpha	UK	Alpha was not associated with an overall increase in mortality among hospitalised patients. However, Alpha may be associated with higher ICU admission and death in females compared with non-Alpha.
Swann, 2021 [36]	Jan 17, 2020 - Jan 31, 2021	medRxiv	To characterise and compare the clinical features and outcomes of children and young people aged <19 years who were hospitalised with SARS-CoV-2 infection during the first and second waves across England, Scotland, and Wales	Hospitals	2044 children and young people aged <19 years were reported from 187 hospitals	Primary outcomes: Admission to critical care; development of Multisystem Inflammatory Syndrome in Children (MIS-C); and in-hospital mortality for children and young people with symptomatic SARS-CoV-2 infection.	Alpha	England, Scotland, and Wales	Severe disease in children and young people admitted with symptomatic SARS-CoV-2 in the UK remains rare. One in five in this cohort had asymptomatic/incidental SARS-CoV-2 infection. No evidence of increased disease severity was found in wave 2 compared with wave 1.
Takemoto, 2021 [51]	Mar 2020-Apr 12th, 2021	medRxiv	To examine impact of Gamma variant on obstetric population in Brazil	Community	8,248 COVID-19 maternal SARS cases	Confirmed maternal deaths in women 10-50 years old	Gamma	Brazil	The number of COVID-19 maternal deaths increased significantly (7.4% vs 15.6%) from 2020 to 2021 in Brazil.
Twohig, 2021 [69]	Mar 29 - May 23, 2021	Lancet Infectious Diseases	To characterise the severity of the delta variant compared with	Community	43,338 COVID-19-positive patients (8,682	The risk for hospital admission and	Alpha, Delta	UK	There was higher hospital admission or emergency care

Author, date	Date of data collection	Source	Objective	Setting	Sample size	Outcome measures	Variant	Country	Main finding
			the Alpha variant by determining the relative risk of hospital attendance outcomes		with the delta variant, 34,656 with the alpha variant)	emergency care attendance			attendance risk for patients with COVID-19 infected with the Delta variant compared with the Alpha variant.
Vassallo, 2021 [37]	Oct 2020-Apr 2021	Journal of Clinical Medicine	To collect the clinical and demographic characteristics of patients admitted to a medical department for COVID-19 due to Alpha and to compare these data with those of patients admitted for COVID-19 due to wild-type during the previous wave	Hospital	158 patients (65 with Alpha, 93 with wild-type)	Clinical characteristics (e.g., admission to ICU, death) and severity of disease, measured by a list of laboratory markers	Alpha	France	Alpha had 3.8-fold higher risk of death or transfer to the ICU compared to wild-type.
Veneti, 2021 [70]	Dec 28, 2020 - June 8, 2021	PLOS One	To estimate the risk of hospitalisation and ICU admission for Alpha and Beta in Norway, compared to non-VOC	Community	28,301 known VOC (Alpha or Beta) or non-VOC cases were included in the analysis (of an original 65,040 identified cases)	Rate of hospitalization and ICU admission	Alpha, Beta	Norway	There is a higher risk of severe disease among people infected with Alpha or Beta, compared to non-VOC
Veneti, 2021 [71]	May 3 2021 - August 15 2021	medRxiv	To estimate the risk of hospitalisation among reported cases of Delta compared to Alpha in Norway	Community	7,977 cases of Delta and 12,078 cases of Alpha (20,055 total)	Rate of hospitalization, vaccination status	Alpha, Delta	Norway	No observed difference in risk of hospitalization between Delta versus Alpha cases.
Whittaker, 2021 [72]	Dec 21st 2020 - April 25th 2021	Journal of Infection	To compare the trajectory of hospitalized patients with Alpha compared with other variants	Hospital	1,186 patients (946 Alpha, 157 non-VOC, 27 (Beta, Gamma, & Kappa), 53 could not be distinguished)	Length of stay in hospital and ICU, mortality	Alpha, Beta, Gamma	Norway	No difference was found in the time from symptom onset to hospitalization, length of stay in hospital and ICU, nor odds of mortality up to 30 days post-discharge for persons infected with Alpha compared with non-VOC.
Zavascki, 2021 [38]	June 2020 - May 2021	Research Square	We investigate the requirement of ventilatory support and mortality rates in non-elderly adult patients	Hospital	In the main cohort a total of 86 (43 Gamma and 43 non-Gamma)	The primary outcome was the incidence rate of need of advanced ventilatory support. Also	Gamma	Brazil	In non-elderly hospitalized patients, COVID-19 caused by Gamma VOC may present a more severe

Author, date	Date of data collection	Source	Objective	Setting	Sample size	Outcome measures	Variant	Country	Main finding
			hospitalized due to COVID-19 caused by Gamma and non-Gamma SARS-CoV-2 lineages		patients were included. The sensitivity cohort included 433 patients: 259 from the first and 174 from the second period (before and after the emergence of Gamma, respectively).	reported on 28-day mortality			clinical course, with increased need of advanced respiratory support and higher 28-day mortality.
Zhao, 2021 [78]	Sep 1st, 2020-Jan 31st, 2021	Viruses	To assess the risk of COVID-19 case fatality using the disease surveillance data to reconstruct the real-time and variant-specific CFR of COVID-19 and infer the change in fatality risk associated with Alpha	Community	149,789 complete human SARS-CoV-2 strains from the GISAID	CRF	Alpha	UK	Overall CFR increased from 1% in September 2020 to 2.2% in November 2020 and stabilized at this level. The variant-specific change in CFR may increase by 18% of fatality risk compared with the wild-type.

CFR = case facility ratio; VOC = variants of concern; SGFT = S-gene target failure; ICU = intensive care unit; UK = United Kingdom; PCR = polymerase chain reaction; HCW = healthcare worker; PPE = personal protective equipment; ARF = acute respiratory failure
